# Two Classes of Protein Therapeutics: Why Dose–Response Architecture Defines the Boundary of mRNA Medicines

**DOI:** 10.3390/pharmaceutics18080941

**Published:** 2026-07-30

**Authors:** Sarfaraz K. Niazi

**Affiliations:** College of Pharmacy and Pharmaceutical Sciences, Washington State University, Spokane, WA 99202, USA; s.niazi@wsu.edu

**Keywords:** mRNA therapeutics, exposure-controlled therapeutics, threshold-response therapeutics, dose–response variability, therapeutic index, translational amplification, threshold pharmacology, lipid nanoparticles, immune-silent delivery, genome editing, protein replacement, indication triage

## Abstract

Messenger RNA (mRNA) entered clinical medicine through vaccines, where the innate immune reactivity that complicates protein therapeutics acts as a built-in adjuvant. The success of the coronavirus disease 2019 (COVID-19) mRNA vaccines inspired an expansive vision of an mRNA 2.0 era extending the modality to rare and common diseases, with delivery framed as the principal challenge. This review accepts much of that vision but argues it rests on an unstated assumption: that all protein therapeutics form a single pharmacological class. They do not. We propose a taxonomy, the Dose–Response Architecture Classification, separating two classes. Exposure-controlled therapeutics require a specific quantity of active protein on a defined regimen, as outcomes depend on reproducible exposure; examples include insulin, erythropoietin, growth hormone, coagulation factors, and narrow-therapeutic-index biologics. Threshold-response therapeutics depend on surpassing a functional threshold rather than maintaining a precise concentration; these include vaccines, many enzyme-replacement therapies, genome editing, receptor-saturating antibodies, and immune-cell reprogramming. Because an mRNA drug is dosed as an instruction, and a single message is translated into a variable number of proteins through a multiplicative, stochastic intracellular chain, the dose-to-effect relationship is inherently variable. Our central, deliberately falsifiable proposition is that mRNA is suitable for threshold-response therapeutics but unsuitable for exposure-controlled therapeutics unless a construct or delivery system demonstrates validated post-delivery output control within predefined pharmacokinetic and pharmacodynamic limits. This is a pharmacological boundary, not a delivery obstacle. We conclude that the greatest advances in mRNA 2.0 will come not from delivery alone but from disciplined indication triage by class.

## 1. Introduction: Why a Version Number

Few biomedical technologies have transitioned from laboratory curiosity to worldwide deployment as swiftly as messenger RNA. Over the course of three decades following the demonstration that exogenous mRNA could direct protein synthesis in vivo [1,2], this approach remained promising yet marginal, impeded by two fundamental challenges: synthetic mRNA is identified by the innate immune system as a pathogen-associated molecule, and it is rapidly degraded by endogenous nucleases. The coronavirus disease 2019 (COVID-19) vaccines, tozinameran (Comirnaty) and elasomeran (Spikevax), effectively addressed these issues, thereby enabling their development as vaccines and, in the process, facilitating the administration of billions of doses globally [3,4,5]. This advancement has sparked discussions on a sequential development of the technology: a first-generation that validates the concept, followed by subsequent generations that significantly broaden the product’s applications beyond its initial purpose [6]. This review accepts that trajectory but argues that it rests on an unstated assumption, namely that all protein therapeutics behave as a single pharmacological class. The organizing contribution offered here, introduced at the outset so that the argument can be read against it, is the Dose–Response Architecture Classification. It separates exposure-controlled therapeutics, whose benefit requires a defined quantity of active protein on a fixed regimen, from threshold-response therapeutics, whose benefit requires only that a functional threshold be exceeded. The central proposition, developed in Section 4 and applied as a triage rule in Section 14, is that mRNA is suited to the second class and, absent validated post-delivery output control, unsuited to the first. This is a pharmacological boundary rather than a delivery obstacle.

The version number specified in this review indicates a clear and definitive progression. mRNA 1.0 represents the immunostimulatory vaccine model, distinguished by the mRNA’s innate immune reactivity and the inaugural lipid nanoparticle (LNP) technology. This reactivity is regarded not as a defect but as an advantage, serving as a self-adjuvant that bolsters the adaptive immune response elicited by the vaccine [7]. The vaccination protocol primarily comprises an initial dose followed by a limited series of booster doses, with administration generally conducted via the intramuscular route. The transient expression of mRNA is negligible, as the adaptive immune system establishes enduring immunological memory. Conversely, mRNA 2.0 embodies the therapeutic approach, wherein the aim is to ensure that the cellular machinery reads the message in a subdued manner, synthesizes a functional protein, antibody, genome editor, or chimeric receptor, and minimizes inflammatory signals that could potentially harm the patient upon recurrent exposure or impair the translation of the therapeutic agent itself [8,9].

This article traces that transition, comparing versions 1.0 and 2.0 throughout and treating the prevailing optimism as a central consideration rather than a caveat. It moves from the molecular anatomy of an mRNA drug and its five optimization levers to the dose-control constraint that most analyses undervalue to lipid nanoparticle delivery and the four frontiers that separate the eras. Clinical evidence across the major therapeutic classes is then re-examined by asking not only whether mRNA can express the relevant protein but also whether the dose–response architecture of the indication tolerates the variability of expression. The account closes with the convergence thesis: mRNA 2.0 is best understood not as a universal protein-manufacturing platform but as a convergence technology, accelerated by artificial-intelligence-guided design and next-generation delivery, whose credible scope is set by honest triage of indications.

A brief biological orientation is useful. Messenger RNA (mRNA) is deliberately transient within the central dogma: it carries instructions from the genome to the ribosome and is then degraded, so an mRNA therapeutic alters cellular function without changing the identity of the cell. It does not cross the nuclear membrane, integrate into the genome, or produce heritable change. The exception is a genome-editing payload, where the mRNA still does not integrate but encodes a protein that enters the nucleus and deliberately modifies DNA, as discussed below. This transience underpins both the modality’s safety profile and its central engineering problems. Unlike small-molecule drugs, mRNA therapies are not active agents but instructions that direct the patient’s own cells to make the therapeutic product, whether a missing enzyme, an antibody, a genome editor, or a chimeric antigen receptor. Two objectives follow: deliver the instruction to the intended cells without provoking an adverse immune response and control the magnitude and duration of expression.

It is also imperative to highlight the significance of this distinction, given its substantial implications for commerce and public health, extending well beyond mere theoretical discourse. The diseases targeted by mRNA 1.0 are, by definition, those for which the primary objective is to provoke an immune response. Conversely, the diseases targeted by mRNA 2.0 include commonplace cardiovascular and metabolic disorders, a wide array of individually rare genetic diseases lacking traditional, economically viable pharmacological treatments, and autoimmune conditions where current therapies tend to broadly suppress immunity rather than target specific pathways. If the platform can be reliably developed to deliver functional proteins and perform precise, safe, and repeatable genetic modifications, the potential patient cohort would increase exponentially beyond those eligible for vaccination. This fundamental capability is the principal reason the field considers this transition a new era rather than merely an incremental development. The argument for dosing precision, central to this review, builds on the author’s previous framework paper [10], and the current article expands on that work rather than merely reiterating it. The novel contribution here is the two-class Dose–Response Architecture Classification, presented as an explicit framework with operational criteria for assignment, encompassing a broader clinical triage across enzyme replacement, genome editing, antibody, cancer-vaccine, and regenerative indications, with updated and timestamped pipeline evidence, along with new figures illustrating the relationship between output variability and dose–response architecture.

### Scope, Sources, and Preparation Methods

This is a narrative review rather than a systematic one. Literature was identified through PubMed, Scopus, and Web of Science covering the period from 1990 through July 2026, using combinations of the terms messenger RNA therapeutics, lipid nanoparticle, dose–response, therapeutic index, protein replacement, base editing, and self-amplifying RNA, supplemented by citation tracking from the retrieved reviews. Clinical and pipeline status were verified against ClinicalTrials.gov registry entries and company disclosures accessed on 23 July 2026.

**Figure 1:**
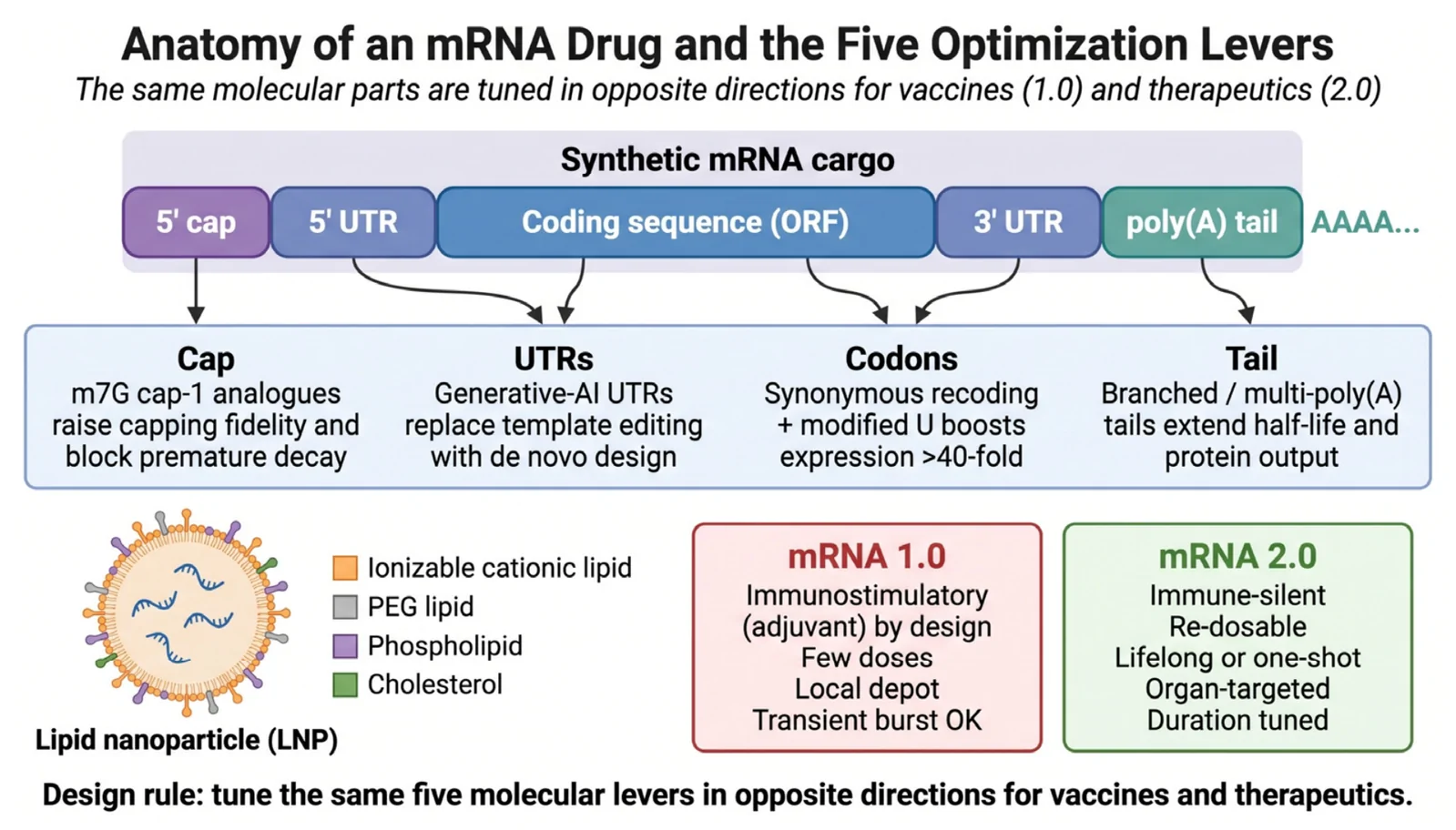
The molecular architecture of an mRNA therapeutic and the five optimization levers. A synthetic mRNA consists of a 5′ cap, a 5′ untranslated region (UTR), the coding sequence, a 3′ UTR, and a polyadenylated tail, each delivered within a lipid nanoparticle (LNP) composed of an ionizable cationic lipid, a PEG lipid, a phospholipid, and cholesterol. The five levers, namely capping, UTR design, codon optimization, nucleoside modification, and poly(A) engineering, are adjusted inversely for vaccines (mRNA 1.0) and therapeutics (mRNA 2.0). The contrasting panels illustrate this inversion: a vaccine is inherently immunostimulatory, administered in a few localized doses with transient expression that the adaptive immune response amplifies, whereas a therapeutic must be immune-silent, re-dosable or single-shot, organ-targeted, and calibrated for the desired duration. Created in BioRender. Niazi, S. (2026) https://BioRender.com/5ooptz1 (Accessed on 10 July 2026).

**Figure 2:**
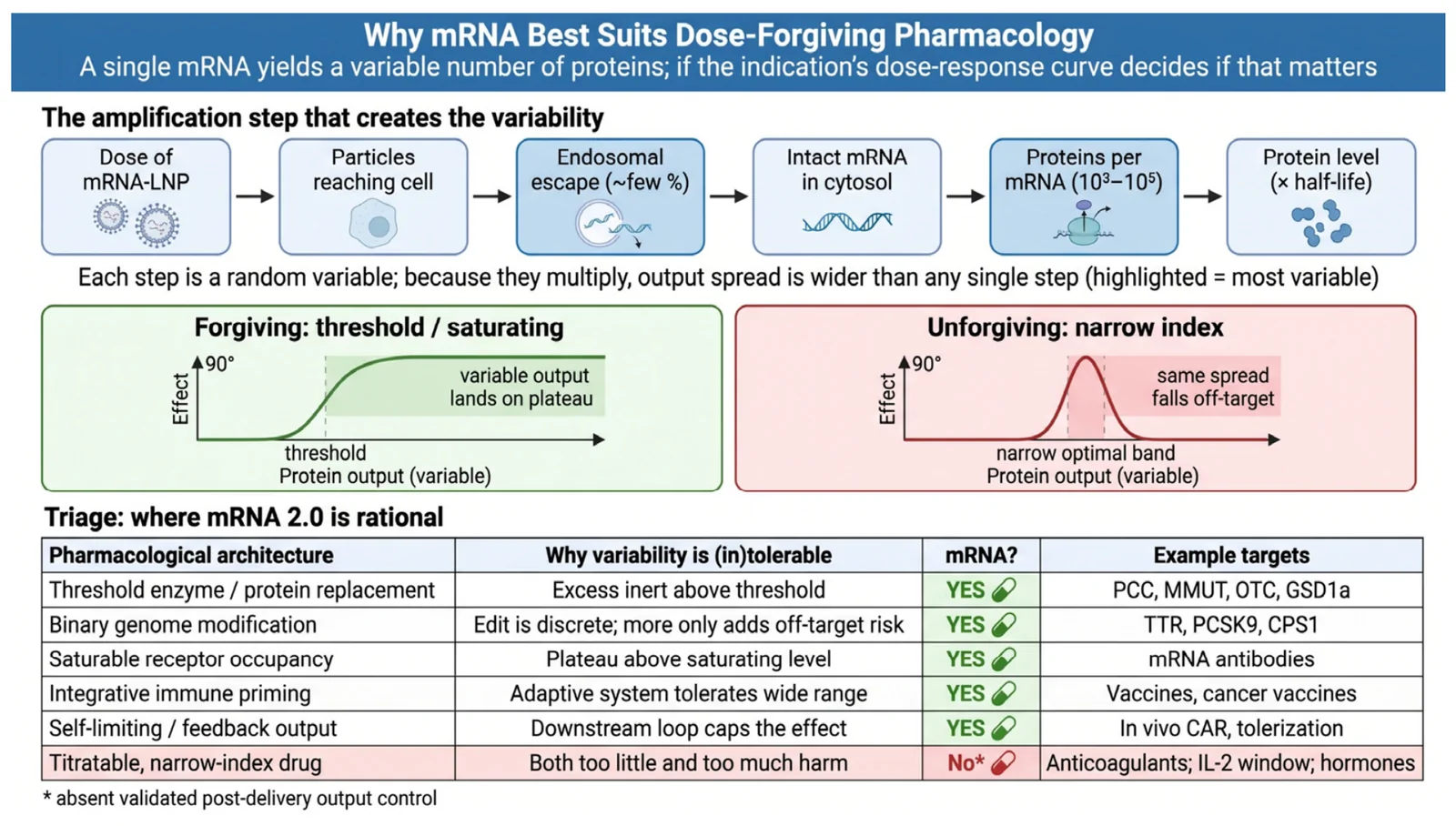
Rationale behind the tolerability of mRNA output variability in specific dose–response architectures. The top panel illustrates that the conversion process from an administered mRNA dose to functional protein involves a series of multiplicative stochastic steps, resulting in an output distribution that exceeds the variability observed in any individual step. The middle panel demonstrates that when the dose–response curve is either threshold-limited or saturating, the resulting variable output resides on a flat plateau, rendering the variability clinically insignificant (left). Conversely, when the curve is narrow and bidirectional, the same variability extends beyond the optimal range, potentially leading to subtherapeutic or toxic outcomes (right). The bottom panel presents a classification of pharmacological architectures: threshold replacement, binary genome modification, saturable receptor occupancy, integrative immune priming, and self-limiting or feedback-regulated outputs are tolerant to variability and inform the rational scope of mRNA 2.0. Conversely, titratable drugs with a narrow therapeutic index are not tolerant of such variability and should be avoided unless closed-loop output control mechanisms are implemented. Created in BioRender. Niazi, S. (2026) https://BioRender.com/5ooptz1 (Accessed on 10 July 2026).

**Figure 3:**
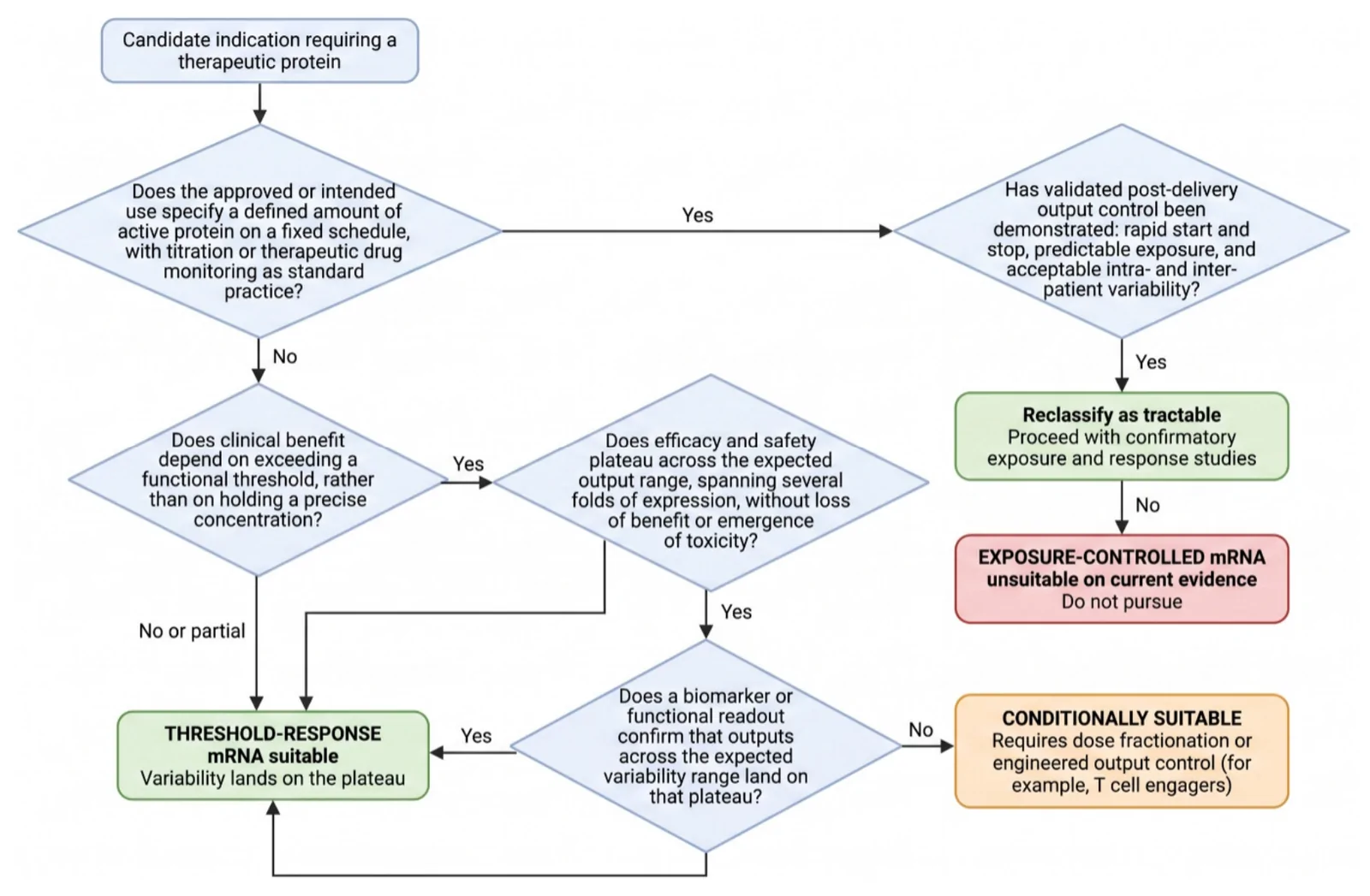
An operational decision flowchart for assigning a candidate indication to the Dose–Response Architecture Classification. The sequence applies the assignment criteria defined in Section 4.2. An indication is exposure-controlled when its approved or intended use specifies a defined quantity of active protein on a fixed schedule with titration or therapeutic drug monitoring, or when a benefit depends on holding a concentration rather than exceeding a threshold. It is a threshold-response indication when benefit follows from exceeding a functional threshold, when efficacy and safety plateau across several folds of expression, and when a biomarker or functional readout confirms that outputs across the expected variability range land on that plateau. Indications whose upper bound sits close to the effective level, such as potent T cell engagers, are conditionally suitable and require dose fractionation or engineered output control. An exposure-controlled indication is reclassified as tractable only where validated post-delivery output control has been demonstrated, which is the falsifiable condition stated throughout this review. Created in BioRender. Niazi, S. (2026) https://BioRender.com/5ooptz1 (Accessed on 10 July 2026).

**Figure 4:**
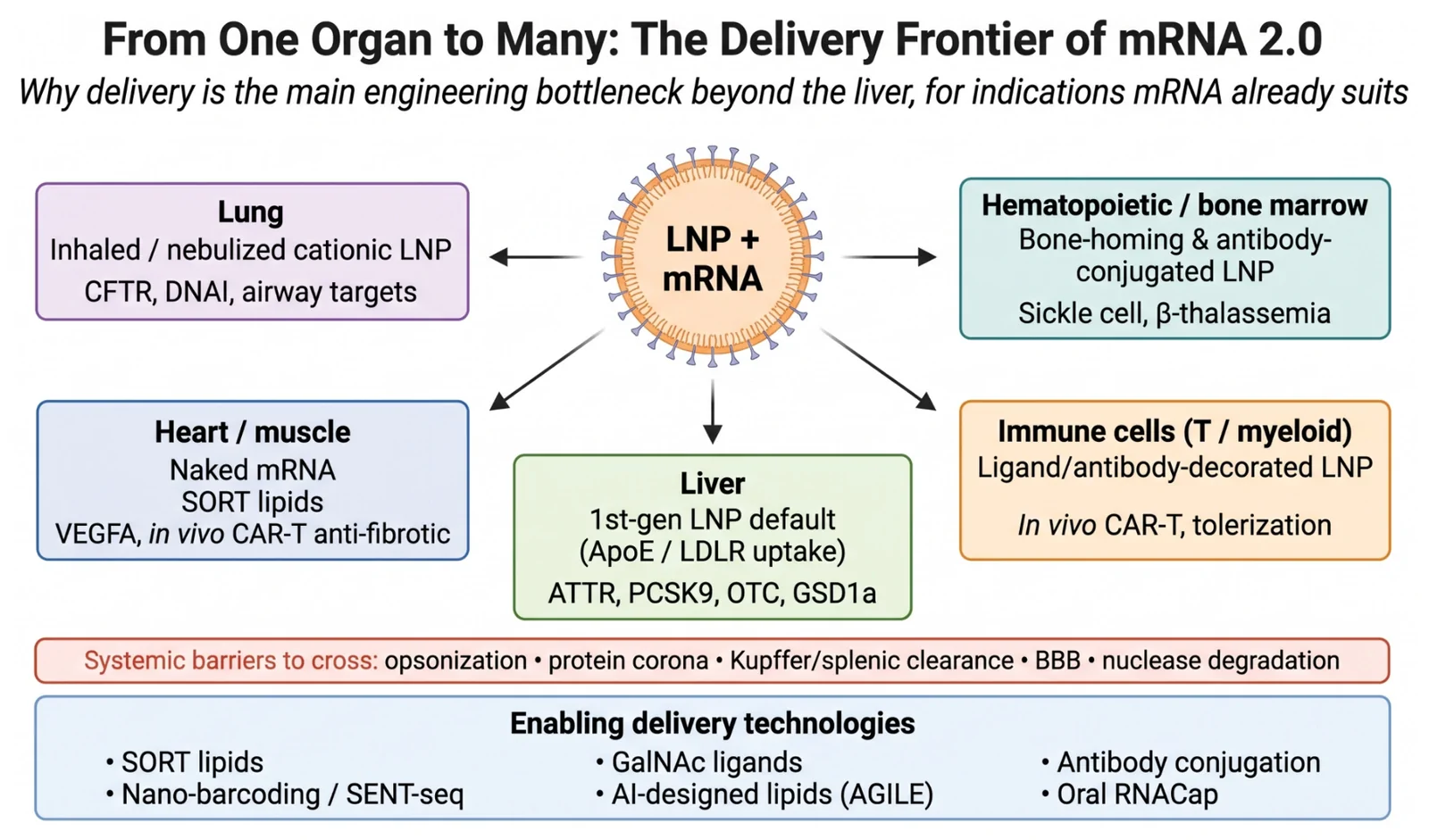
The delivery frontier of mRNA 2.0. First-generation lipid nanoparticles (LNPs) predominantly target the liver through apolipoprotein-mediated, receptor-driven uptake, which is advantageous for hepatic applications such as transthyretin amyloidosis, PCSK9, and ornithine transcarbamylase deficiency; however, this approach limits further expansion. Achieving delivery to the heart, muscle, lung, hematopoietic, and bone marrow compartments, as well as specific immune cell subsets, necessitates the development of engineered delivery methods, including SORT lipids, ligand- and antibody-conjugated particles, inhaled cationic formulations, and bone-homing chemistries. The systemic barriers encountered by any intravenous particle, along with the emerging enabling technologies, are outlined. For pharmacologically suitable threshold-response indications, the overarching message of this era is that delivery, rather than the therapeutic message itself, constitutes the primary engineering bottleneck, particularly beyond hepatic targets; however, delivery does not extend mRNA to exposure-controlled indications, whose suitability is determined by dose–response architecture rather than by reach. Created in BioRender. Created in BioRender. Niazi, S. (2026) https://BioRender.com/5ooptz1 (Accessed on 10 July 2026).

**Figure 5:**
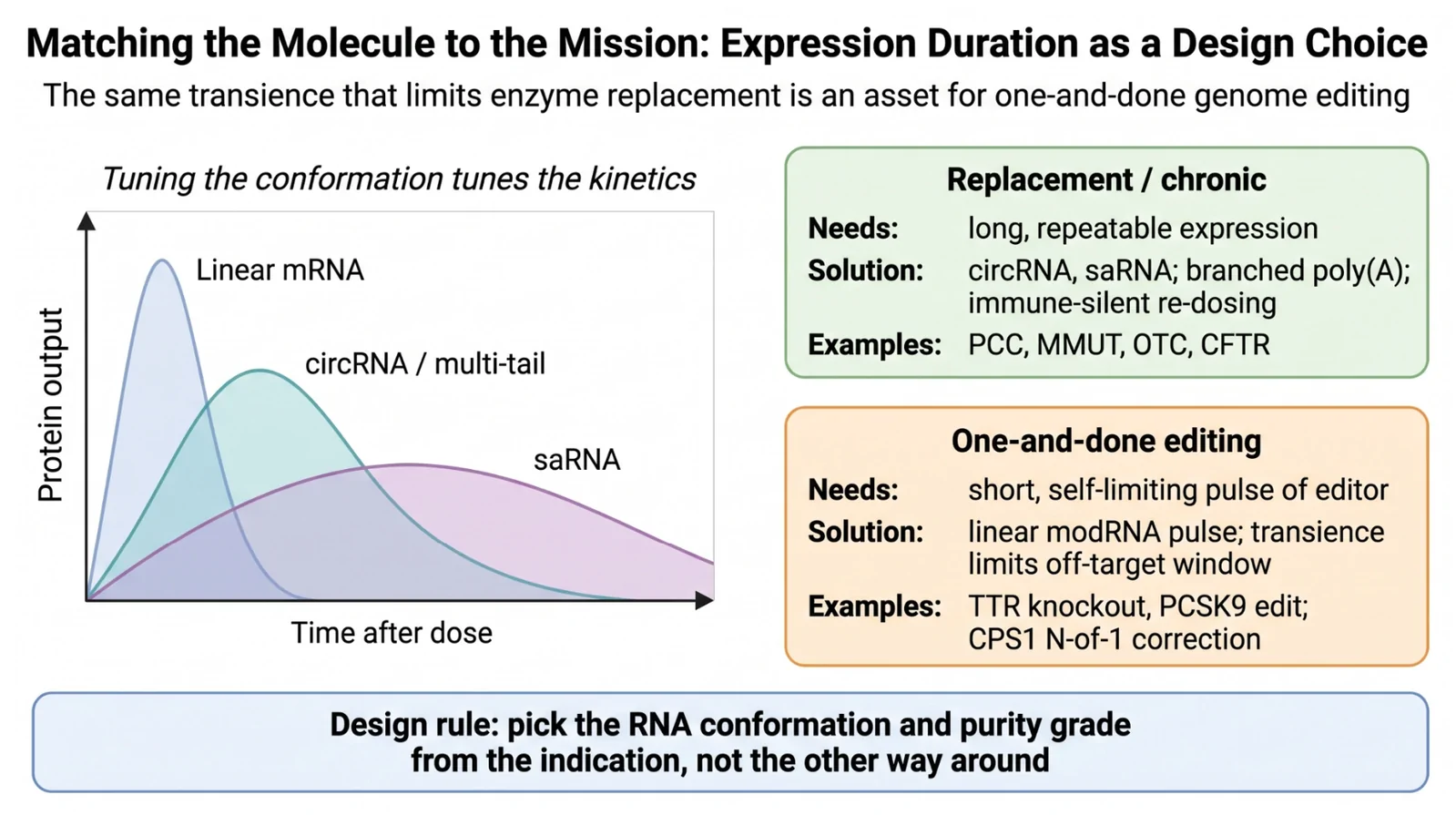
Expression duration as a design choice. Linear modified mRNA produces a rapid, short-lived pulse of protein; circular and multi-tailed mRNAs broaden and sustain expression; and self-amplifying RNA results in a delayed, high-amplitude, prolonged output. The right panels map these kinetics onto two therapeutic logics. Replacement and chronic indications (for example, propionic acidemia, methylmalonic acidemia, ornithine transcarbamylase deficiency, and cystic fibrosis) require long-lasting, repeatable expression and immune-silent re-dosing. Narrow-window immunomodulators such as IL-2 muteins are deliberately excluded here, as their bidirectional dose sensitivity places them in the exposure-controlled class that requires output control rather than in the chronic-replacement category. One-and-done editing indications (transthyretin knockout, PCSK9 base editing, and the CPS1 N-of-1 correction) capitalize on transience, as a self-limiting pulse of the editor reduces the off-target window. Created in BioRender. Created in BioRender. Niazi, S. (2026) https://BioRender.com/5ooptz1 (Accessed on 10 July 2026).

Generative artificial intelligence was used in the preparation of this manuscript in two defined roles: to search the literature to surface candidate references, every one of which the author then retrieved and verified against the primary source, and to correct grammar and language. No generative artificial intelligence tool was used to generate or analyze data, to design the review, to produce any scientific claim or interpretation, or to formulate the Dose–Response Architecture Classification, its assignment criteria, or the triage rule derived from it, all of which are the author’s own work. The author verified every reference, every quantitative statement, and every figure against its source and takes full responsibility for the content of the manuscript. The author used BioRender to create Figure 1, Figure 2, Figure 3, Figure 4 and Figure 5, for which licenses are shared; a link to the license is included in the figure description. https://app.biorender.com/citation/6a473f90c2a11875b12f21dd (accessed on 10 July 2026).

## 2. The Molecular Anatomy of an mRNA Drug

A synthetic messenger RNA (mRNA), generated through in vitro transcription, constitutes a single-stranded molecule comprising five functional components: a 5′ cap, a 5′ untranslated region (UTR), the coding sequence or open reading frame (ORF), a 3′ UTR, and a polyadenylated (poly(A)) tail (see Figure 1). Each component has undergone extensive optimization, and the aggregate effect of these improvements has been instrumental in rendering the product clinically viable [11,12]. A comprehensive understanding of these five regulatory elements is critical, as the distinction between a first-generation (1.0) and an advanced (2.0) construct is seldom a matter of different parts; rather, it concerns how these identical parts are precisely calibrated and tuned (Figure 1).

### 2.1. The 5′ Cap

The 5′ cap comprises a 7-methylguanosine (m7G) moiety attached via a triphosphate linkage to the initial transcribed nucleotide. It is crucial for the initiation of translation, functions to safeguard the transcript from exonucleolytic degradation, and assists in differentiating self from non-self RNA during innate immune surveillance [13,14,15]. The first generation of synthetic caps, exemplified by the anti-reverse cap analog (ARCA) designed to prevent incorporation in the reverse orientation, achieved a capping efficiency of approximately 70 percent and remained vulnerable to premature degradation [16]. Subsequent developments have led to the creation of co-transcriptional cap-1 analogs with capping efficiencies approaching 95% and improved resistance to decapping, collectively enhancing protein yield and reducing immunogenicity [17,18,19]. These advanced cap chemistries are incorporated in the approved COVID-19 vaccines as well as the self-amplifying vaccine zapomeran (Kostaive) [20].

### 2.2. The Untranslated Regions

The 5′ and 3′ untranslated regions (UTRs) do not encode proteins; however, they play a crucial role in regulating gene expression. The 5′ UTR primarily governs the initiation of translation, whereas the 3′ UTR predominantly influences transcript stability and half-life [21,22]. Traditionally, UTR optimization involved introducing variants into known natural sequences and empirically screening them. The advent of deep learning models has revolutionized this process, shifting the focus from editing existing templates to de novo design. UTRGAN was the pioneering generative framework that produced synthetic 5′ UTR sequences optimized for specific target properties [23]. Concurrently, 3′ UTR optimization has progressed through three parallel strategies: AI-guided de novo synthesis [24,25], cell-type-specific combinatorial screening [26], and programmable modulation of RNA-binding proteins [27]. The capacity to generate customized UTRs for any given gene constitutes a foundational element of tissue-specific design 2.0 [28].

### 2.3. Codon Optimization and Nucleoside Modification

The coding sequence functions as a regulatory hub as much as an instruction set. Codon optimization, referring to the substitution of synonymous codons without altering the encoded protein, enhances ribosome engagement and elongation, thereby increasing expression and stability [29,30,31]. Current methodologies prioritize uridine depletion and elevated GC content to prevent recognition by the cytosolic sensor RIG-I. The most significant chemical advancement, however, was the discovery by Kariko and Weissman that incorporating modified nucleosides, particularly pseudouridine and subsequently N1-methylpseudouridine, markedly reduces activation of Toll-like receptors TLR3, TLR7, and TLR8, as well as RIG-I, while concurrently enhancing translation. Innate sensing is not governed by nucleoside identity alone; it also depends on the 5′-triphosphate moiety, double-stranded RNA impurities, secondary structure, dose, and cell type, so modified nucleosides attenuate rather than abolish recognition [32,33,34,35]. N1-methylpseudouridine increases ribosome density and pausing in a manner that elevates output [36] and diminishes innate sensing [37]. Codon optimization and modified uridine each increase protein output, and reported gains vary widely across constructs, cell types, and comparators, rather than converging on a single universal factor. In an early and frequently cited comparison, nucleoside-modified, HPLC-purified mRNA increased reporter-protein expression in mammalian cells and in mice by roughly an order of magnitude or more relative to unmodified, unpurified transcript, though the exact fold-change is construct- and context-dependent and should not be read as a fixed constant [38,39]. This combined approach is now employed in many approved COVID-19 mRNA vaccines and numerous clinical-stage therapeutics. More recently, generative artificial intelligence frameworks such as GEMORNA and CodonBERT have advanced beyond predefined sequence features to incorporate the contextual complexity of codon usage and directly predict translational efficiency [40,41]. Broader multimodal foundation models spanning DNA, RNA, and protein tasks sit alongside these codon-specific tools and extend the same design logic to a wider range of sequence properties, although they do not themselves address codon optimization [42].

### 2.4. The Poly(A) Tail

The poly(A) tail confers stability to the transcript and enhances translation by facilitating a closed-loop interaction between the 5′ cap and poly(A)-binding proteins, thereby protecting the message from exonucleolytic degradation [43,44]. The engineering of the tail, whether by optimizing its length, incorporating structural elements such as poly(U) segments or upstream stem-loops, or employing branched and multi-tailed architectures, modulates both stability and protein output [45,46]. Chemical modifications at the tail terminus and the adoption of branched poly(A) configurations further augment and extend protein production [47,48,49]. Collectively, the elements comprising the cap, untranslated regions (UTRs), a codon-optimized open reading frame (ORF), and a poly(A) tail enable a modular approach in which each component is strategically selected to optimize stability and translational efficiency for specific applications [31,50]. The poly(A) tail is central to this behavior rather than incidental: its length and integrity set transcript half-life and translational output by nucleating the closed-loop interaction between the 5′ cap and poly(A)-binding proteins, by shielding the message from 3′ to 5′ exonucleolytic decay, and by tuning the timing of deadenylation-triggered turnover. In practice, tail length and terminal chemistry are primary levers for matching expression duration to the indication, which is why tail-length distribution is treated as a release-defining quality attribute for therapeutic mRNA.

None of these five levers has a single correct setting. A vaccine and an enzyme-replacement therapy may share the same cap chemistry yet require different expression durations and immune profiles; therefore, the cargo is best conceptualized as a configurable toolbox rather than a fixed molecule.

### 2.5. Innate Sensing as the Unifying Constraint

A single immunological mechanism unites the five levers and explains why one chemical insight reshaped the field. Mammalian cells carry a network of pattern-recognition receptors that detect foreign nucleic acids. Specifically, the endosomal Toll-like receptors TLR3, TLR7, and TLR8 detect double- and single-stranded RNA, whereas the cytosolic sensors RIG-I and MDA5 recognize double-stranded and 5′-triphosphate RNA. Additionally, protein kinase R and the 2′-5′-oligoadenylate synthetase system respond to double-stranded species by globally inhibiting translation [32,37]. Unmodified synthetic mRNA can activate multiple of these sensors simultaneously, resulting in both cellular injury and, via the kinase-R pathway, the suppression of translation of the very message that was delivered. The groundbreaking discovery by Kariko and Weissman that substituting uridine with pseudouridine or N1-methylpseudouridine substantially attenuates this recognition is therefore not merely a marginal refinement. Instead, it marked the pivotal event that transformed mRNA from an inflammatory liability into a viable therapeutic agent. The attenuation is not absolute: residual sensing persists in proportion to 5′-triphosphate content, double-stranded RNA contamination, construct structure, and dose, which is why high-fidelity capping and ultra-purification remain necessary alongside nucleoside modification [34,35]. All other strategies, such as high-fidelity capping, uridine-depleted codon selection, GC content optimization, and ultra-purification to remove double-stranded byproducts, serve the same overarching purpose of ensuring that the construct remains beneath the innate immune detection threshold.

The immunological consequence is asymmetric. A vaccine can tolerate, and indeed exploit, residual innate activation because the resulting interferon and cytokine signals act as adjuvants that enhance the adaptive response. Conversely, a therapeutic cannot, as these signals are either directly toxic at the higher systemic doses required or actively undermine the therapy by halting translation. The entire chemical and manufacturing program of mRNA 2.0 can be interpreted as the pursuit of a construct that is simultaneously highly translatable and immunologically silent, two properties that are inherently conflicting and must be carefully balanced rather than maximized independently [31,50].

## 3. The Delivery Vehicle and the Tyranny of the Liver

The efficacy of an mRNA pharmaceutical depends on its delivery system. The lipid nanoparticle (LNP), which safeguards the messenger RNA (mRNA), facilitates its transit across the cellular membrane via an endosomal pathway and releases it into the cytoplasm, constitutes a critical component fundamental to the development of all approved mRNA vaccines [51,52,53]. A standard LNP comprises four lipid constituents: an ionizable cationic lipid, a PEGylated lipid, a phospholipid, and cholesterol. The ionizable lipid, which binds and stabilizes the mRNA and promotes endosomal escape, is regarded as the most essential element. Clinically validated examples include SM-102 (utilized in elasomeran), ALC-0315 (employed in tozinameran), and the analogous chemistry used in the initial approved small interfering RNA (siRNA) therapy, patisiran [54,55].

Although LNPs dominate clinical practice, non-lipid carriers are advancing and merit acknowledgment. Polymeric systems based on poly(beta-amino ester)s, polyethylenimine derivatives, and charge-altering releasable transporters offer tunable degradation and, in some designs, intrinsic tropism away from the liver. Lipopolyplexes and polymer-lipid hybrids combine polymeric condensation with lipid-mediated endosomal escape. Peptide-based carriers, including cell-penetrating and amphipathic peptides, and protein-based or virus-like particle systems that package transcripts through engineered RNA-binding interactions, provide alternative routes to cytosolic delivery. Inorganic and hybrid scaffolds, exosome- and extracellular-vesicle-derived carriers, and dendrimer-based ionizable materials are also under evaluation. These platforms remain earlier in development than LNPs and face their own manufacturing, purity, and immunogenicity questions, but they broaden the design space for extrahepatic and repeatedly dosed applications.

The primary limitation of first-generation lipid nanoparticle (LNP) systems is their propensity to accumulate in the liver. Once an LNP-encapsulated messenger RNA (mRNA) enters systemic circulation, it must overcome numerous biological barriers, including opsonization, protein corona formation, phagocytic clearance by Kupffer cells and splenic antigen-presenting cells, and renal filtration, before reaching its designated target organ [56,57,58]. First-generation LNPs are efficiently internalized by hepatocytes because they are recognized as lipoproteins and are cleared via well-characterized receptor-mediated endocytic pathways. Furthermore, the fenestrated architecture of the hepatic vasculature allows passive entry into the liver parenchyma [59,60]. Although this hepatic tropism facilitates liver-targeted therapies, it poses a significant challenge for the treatment of other organs, such as the heart, kidney, muscle, lung, or hematopoietic system. As outlined in the comprehensive review framing this research area, the inability to achieve efficient in vivo delivery to organs other than the liver remains the most substantial barrier to expanding the utility of mRNA therapeutics [6].

A second limitation pertains to immunogenicity upon repeated dosing. Even the most advanced clinical LNP-mRNA formulations cannot entirely evade the stimulation of innate immune signals when administered repeatedly, and these signals function to inhibit the translation of the therapeutic message following its release into the cytoplasm [55,61]. In the context of a vaccine, this is considered acceptable, or even advantageous, as immunostimulation serves as an adjuvant activity, and the administered doses are minimal. Conversely, for chronic protein-replacement therapy given biweekly for a lifetime, such immunogenicity is disqualifying. Furthermore, the magnitude of the problem is dose-dependent: vaccines use small, non-systemic doses, while systemic therapeutics require larger intravenous doses, thereby rendering hepatic Kupffer cell uptake, complement activation, and other safety considerations substantially more significant [6].

Optimizing lipid nanoparticle (LNP) formulations for safe and highly efficient hepatic uptake requires comprehensive research in non-human primate models, given that the fenestrae of human and primate livers are smaller than those in mice and that the longer circulatory transit time in larger animals exacerbates protein corona formation and aggregation [62]. A systematic approach to adjusting the ionizable lipid (including its polar head group, linker, and aliphatic chains), cholesterol content, and PEG concentration is anticipated to produce agents with enhanced therapeutic windows [63,64,65].

### 3.1. The Biophysics of Endosomal Escape

The most inefficient stage in mRNA delivery is endosomal escape, and understanding this process highlights the importance of ionizable lipids. After uptake of a lipid nanoparticle (LNP) via receptor-mediated endocytosis, it is transported into progressively more acidic endosomal compartments. A key feature of an ionizable lipid is its near-neutral charge at physiological pH, which reduces non-specific toxicity and extends circulation time; however, as the endosomal environment becomes more acidic, the lipid becomes protonated and positively charged. The protonated lipid is thought to interact with anionic phospholipids within the endosomal membrane, forming non-bilayer, hexagonal structures that destabilize the membrane and facilitate the escape of a portion of the mRNA cargo into the cytosol before lysosomal degradation occurs [52,54]. Estimates of escape efficiency are sobering, as only a small proportion of internalized mRNA typically reaches the cytosol. This suggests that improvements in escape capacity could directly lead to dose reductions and enhanced safety margins. As a result, targeted chemical evolution and AI-driven design of ionizable lipids, optimizing parameters such as head-group pKa, linker biodegradability, and tail saturation, are among the most active areas of research in the development of second-generation mRNA delivery systems [63,64].

The PEG lipid exemplifies a characteristic 2.0 trade-off. PEGylation stabilizes the particle, regulates its size during formulation, and prolongs circulation by decreasing opsonization; however, it also hinders cellular uptake and endosomal escape. Additionally, it has been associated with rare anaphylactic reactions and accelerated blood clearance upon repeated dosing [66,67]. For a vaccine requiring only a few doses, this concern is minimal; however, for a chronic therapeutic regimen involving repeated doses, it constitutes a significant design constraint. Consequently, polysarcosine and other PEG alternatives are under investigation for re-dosable formulations. Cholesterol content influences membrane fusion and rigidity; reducing its levels has been proposed as a strategy to mitigate default hepatic uptake [6].

### 3.2. The Protein Corona and the Case for Primate Models

Once within the bloodstream, a lipid nanoparticle (LNP) does not present its engineered surface to cells; instead, it exhibits a protein corona, which is a layer of plasma proteins that adsorbs within seconds of administration. The composition of this corona, including apolipoprotein E, influences the receptors engaged by the particle and, consequently, its destination [56,59]. The adsorption of apolipoprotein E significantly explains why first-generation particles are directed towards hepatocytes via the low-density lipoprotein receptor. This has two implications for the design of second-generation particles. Firstly, any strategy aimed at targeting tissues outside the liver must address the corona, which can obscure an engineered ligand; techniques such as covalent conjugation and high-avidity targeting are partial efforts to surpass the default trafficking influenced by the corona [68]. Secondly, due to differences in corona composition and the kinetics of its formation across species, and considering that transit times scale with body size, delivery data obtained from rodents are insufficient predictors for human applications. Consequently, studies involving non-human primates are nearly indispensable for systemic candidates in second-generation development [62]. The overarching principle is that delivery constitutes a systems problem, in which the particle, the corona, the vasculature, and the target receptor must be considered collectively.

### 3.3. Modern Approaches to the Delivery Bottleneck

Because delivery, rather than expression, is the rate-limiting barrier for most non-hepatic targets, several complementary strategies are converging to move mRNA beyond the liver. Selective organ-targeting (SORT) lipids add a charged helper lipid that redirects particles to the lung or spleen; ligand-, peptide-, and antibody-conjugated LNPs engage receptors on defined cell types; reduced-cholesterol and re-dosable, PEG-alternative formulations lower default hepatic uptake; and microRNA-responsive elements restrict translation to the intended tissue. Crossing the blood–brain barrier remains the most demanding case: candidate solutions under investigation include receptor-mediated transcytosis using transferrin-receptor or other brain-endothelial ligands, focused ultrasound with microbubbles to transiently and locally open the barrier, engineered viral-capsid-inspired and peptide-decorated nanoparticles selected for central-nervous-system tropism, and direct intrathecal or intracerebroventricular administration that bypasses the barrier altogether. These approaches remain largely preclinical for mRNA, and each must still contend with the protein corona and with species differences that limit extrapolation from rodents to humans.

## 4. The Dose-Control Problem: An Intrinsic Pharmacological Constraint on mRNA

A fundamental limitation of mRNA therapeutics is often overlooked amid optimistic narratives, including recent discourses on an mRNA 2.0 era. This limitation must be acknowledged before assessing therapeutic applications, as it fundamentally shapes the scientific justification for them. The central issue is as follows: whereas traditional drugs involve direct administration of the active compound, mRNA-based therapies deliver instructions to the patient’s cells to synthesize the active compound. The process from instruction to active agent involves multiple biological steps, each subject to variability and limited controllability. A single mRNA molecule can be translated into many protein molecules, with estimates ranging from approximately 1000 to 1,000,000 copies per mRNA through successive rounds of ribosomal translation. These figures are approximate, order-of-magnitude estimates based on translation rates and half-life models rather than fixed constants, intended to illustrate the scale of amplification rather than to serve as definitive values. This variability emphasizes that amplification is not an intrinsic, fixed property of the molecule. The three-orders-of-magnitude variation reflects the dependence of output on the construct and target, rather than mere measurement noise. For example, a cytokine-encoding message, prone to rapid degradation and feedback mechanisms, may produce only 1000–10,000 proteins, whereas an optimized Cas9-encoding message, with enhanced stability and translational efficiency, may yield 100,000–1,000,000 proteins [10,69,70]. At the kinetic level, optimized constructs incorporating engineered untranslated regions, modified nucleosides such as pseudouridine and N1-methylpseudouridine, and codon optimization can translate at rates of approximately 10–100 proteins per message per minute. These figures are provided as illustrative kinetic ranges rather than as fixed, construct-independent constants. Additionally, chemical and sequence modifications extend the functional half-life from mere minutes in unmodified constructs to 24–72 h, allowing the total output to integrate a variable translation rate over a variable lifespan [31]. Consequently, the amplification factor varies depending on cellular translational state, construct design, delivery context, patient variability, and timing. Accordingly, the same nominal dose can result in a wide range of protein expression among recipients. The implication is that the relationship between the administered dose and the resulting functional protein quantity is inherently more variable in mRNA drugs than in small molecules or recombinant proteins. This variability is not a manufacturing flaw remedied by improved processes; rather, it is an intrinsic characteristic of using translation as the final manufacturing step in the patient, which cannot be precisely measured or corrected prior to protein synthesis.

### 4.1. Why the Dose-to-Effect Chain Is Intrinsically Noisy

It is essential to accurately delineate the process, as the involved variability is multiplicative rather than additive, leading to compounded effects. The progression from an administered dose of an mRNA-lipid nanoparticle to the corresponding amount of functional protein at the target site encompasses at least six stochastic stages. The number of nanoparticles that successfully evade circulation and reach a target cell depends on factors such as opsonization, protein corona formation, and clearance mechanisms. The proportion of nanoparticles internalized by cells is influenced by the local receptor environment. The fraction of nanoparticles that escape the endosome prior to lysosomal degradation is notably small and highly variable, often estimated at only a few percent. The quantity of intact mRNA molecules delivered into the cytosol is affected by nuclease activity along the pathway. The number of protein molecules produced per intact mRNA, referred to as the amplification factor, depends on ribosome availability, competition with endogenous transcripts, the construct’s cap, untranslated regions, codon usage, and the activation of innate sensing pathways that may inhibit translation. Ultimately, the amount of protein present at any given time relies on the half-lives of both the message and the protein. Each stage exhibits its own distribution, and since the final output results from the product of these stages, the coefficient of variation in the overall output exceeds that of any individual stage. Direct single-molecule imaging of translation within living cells has demonstrated that individual mRNA molecules can display significantly different translational efficiencies and can reversibly switch between translating and non-translating states [71]. This directly observed heterogeneity establishes that the amplification factor varies at the single-molecule level within a single cell. The step from this observation to the population-level output distribution presented below is model-based inference rather than a directly measured quantity and is presented as such prior to considering additional variations between cells, tissues, or patients. Furthermore, the amplification factor is influenced by construct chemistry, as modifications such as codon optimization and the incorporation of modified nucleosides can alter per-molecule output by more than fortyfold [33,38]. Additionally, the number of intact messages delivered per particle is not fixed but follows a distribution.

The sources of variability among patients are equally tangible. Hepatic uptake of first-generation nanoparticles is facilitated by apolipoprotein E binding and the low-density lipoprotein receptor; thus, a patient’s lipoprotein profile, receptor expression, and genetic factors influence the extent of nanoparticle delivery to hepatocytes. Innate immune activity varies among individuals and fluctuates with age, inflammation, and prior exposure, thereby affecting the extent to which translation is suppressed. Considerations such as hepatocyte turnover, disease status, and concomitant medications also impact the outcome. Consequently, the same nominal dose can produce significantly different protein outputs across different patients and even within the same patient on different occasions. While this may be of minimal concern for a vaccine, it is critically important for a drug whose efficacy depends on binding to a specific protein.

### 4.2. Two Classes of Protein Therapeutics: Exposure-Controlled and Threshold-Response

The inherent error in adopting an excessively optimistic or pessimistic perspective in the field lies in the assumption that all protein therapeutics constitute a single pharmacological class. Such an assumption is inaccurate. Protein therapeutics can be classified into two fundamentally distinct groups, a division that we propose here as a framework, namely, the Dose–Response Architecture Classification, rather than as an established taxonomy. This division, rather than any intrinsic property of the disease or the protein, determines whether mRNA is an appropriate therapeutic modality. The first category comprises exposure-controlled therapeutics, in which clinical benefit hinges on administering a specified amount of active protein according to a predetermined regimen. In this context, outcomes are correlated with reproducible exposure levels, and both underexposure and overexposure can compromise efficacy or safety. Consequently, dosage becomes an integral component of the therapeutic mechanism. The approved labeling precisely delineates the amount and frequency of administration, given that exposure directly influences the therapeutic effect. Examples include insulin, erythropoietin, growth hormone, coagulation factors, numerous cytokines, immunosuppressants maintained within a narrow trough window, and biologics with a narrow therapeutic index. The second category encompasses threshold-response therapeutics, in which clinical benefit depends on achieving a sufficient level of biological activity rather than maintaining a specific concentration. Once a functional threshold is surpassed, additional protein provides minimal or no benefit, as the system tends to integrate, saturate, self-limit, or convert the signal into a discrete event. This category includes vaccines, many enzyme-replacement therapies, genome editing technologies, receptor-saturating antibodies, regenerative signaling, and various forms of immune-cell reprogramming. As a proposed framework, this classification requires operational criteria to assign an indication to one class or the other. An indication is considered exposure-controlled when the approved or intended use specifies a defined quantity of active protein on a fixed schedule, when clinical outcomes track reproducible exposure such that both underexposure and overexposure diminish efficacy or safety, and when the separation between effective and toxic exposure is narrow, on the order of twofold or less, so that titration and therapeutic monitoring are standard practices. Conversely, an indication is classified as threshold-response when clinical benefit depends on exceeding a functional threshold, when efficacy and safety plateau above that threshold across a wide range, spanning several folds of expression, without loss of benefit or emergence of toxicity, and when biomarker or functional evidence confirms that outputs across the expected variability range all fall within that plateau. This framing overlaps with the established narrow-therapeutic-index concept but is not identical to it: the narrow therapeutic index describes the safety window for a delivered active agent, whereas the present classification additionally considers whether the delivery modality, here, translation within the patient, can reliably place the output within that window. Assignment, therefore, depends on the width of the safety and efficacy plateau, the availability of a biomarker that reports the relevant output, and whether variability can be tolerated or must be controlled, rather than solely on the identity of the disease or the protein.

The significance of mRNA is directly linked to the amplification process previously discussed. The exposure-controlled classification aligns with the regulatory concept of a narrow therapeutic index, characterized by a difference of less than approximately twofold between toxic and therapeutic concentrations, thereby requiring careful titration and vigilance. Such medications depend on low inter-subject variability to ensure efficacy; otherwise, a specific dose may be subtherapeutic in some cases and toxic in others [72,73]. For instance, anticoagulants, insulin titrated to glucose levels, antiarrhythmics, immunosuppressants maintained within a strict trough level, or cytotoxic agents administered near their maximum dose limit are inherently exposure-controlled. Any residual variability of several folds in the administered active-agent quantity renders these agents unsuitable for such regulation. These are precisely the therapeutic agents that mRNA, with translation as its final and uncontrolled manufacturing stage, cannot reliably produce.

The interpretation advanced in this paragraph, and the boundary it draws, is the author’s own proposal rather than a consensus position drawn from the cited sources, which are used here to establish the underlying pharmacological facts rather than this classification. This provides a central, explicitly falsifiable proposition articulated in terms of two classes rather than specific diseases or proteins: mRNA is well-suited for threshold-response therapeutics; however, based on current evidence, it is unsuitable for exposure-controlled therapeutics unless intracellular protein output can be validated as being regulated post-delivery within predefined variability limits. The existing literature on therapeutic mRNA, exemplified by the recent comprehensive report on the mRNA 2.0 era [6], does not distinguish this; the presumption that protein therapeutics form a singular class, with delivery as the main remaining obstacle, persists across various reviews in the field [8,9]. This omission represents a significant weakness because, without this distinction, delivery appears to be the primary remaining challenge, and resolving it could potentially enable the use of mRNA for most protein therapeutics. Nonetheless, this inference is unwarranted. When a therapeutic protein has already received approval with a fixed dose and regimen, it is exposure-controlled, and such a product cannot be replicated with current mRNA constructs, which lack validated mechanisms to control post-delivery protein output. The underlying issue is structural rather than due to a deficiency in any specific formulation, and it would only be surmounted by a construct or delivery system that demonstrably regulates output after delivery. An approval predicated on fixed-dose, fixed-regimen parameters encodes a specific exposure profile: a designated quantity of active agent administered on a specified schedule, with intra-subject variability maintained within approved limits. An mRNA drug cannot duplicate this profile because it does not deliver the active agent directly; instead, it provides an instruction whose conversion into an active agent involves a multiplicative, stochastic, in vivo amplification process, rendering the actual quantity delivered only ascertainable within a broad range and only after protein synthesis has taken place. Advances in capping, untranslated-region design, codon optimization, purification, lipid nanoparticle targeting, duration control, or artificial intelligence-guided design do not resolve this issue, as each narrows one source of variance while leaving the translation step, the final, unmetered process, unchanged. This challenge extends beyond mere delivery considerations. Delivery determines mRNA localization, whereas pharmacology determines the suitability of mRNA utilization. The fundamental assertion that the field must accept is that, without validated post-delivery output regulation, current mRNA platforms cannot fulfill an exposure-controlled therapeutic role, whether by replacing an existing fixed-dose product or by enabling a new one. This statement is intentionally falsifiable: a construct demonstrating rapid start-and-stop control, predictable exposure, and clinically acceptable intra- and inter-patient variability would satisfy this criterion. Its primary domain resides within the threshold-response class, where the biology is indifferent to the precise amount produced.

The threshold-response class is not monolithic, and understanding its internal structure clarifies why it is extensive and significant rather than merely a niche exception. It consists of five pharmacological architectures. The first involves threshold-limited enzyme replacement, whereby, for many but not all deficiencies, restoring even a fraction of normal enzymatic activity eliminates the disease phenotype, and additional activity is metabolically silent because the enzyme operates well below saturation on an accumulating substrate; in these cases, any output exceeding the threshold is therapeutic, and excess does no harm, rendering variability in the upward direction inconsequential. This tolerance is disease-specific rather than a universal property of the class. It does not apply where excess enzymatic activity, expression in inappropriate tissues, incorrect subcellular localization, immune response to the expressed protein, or depletion of a necessary substrate could be harmful, and such targets instead behave as exposure-controlled and require the output regulation discussed below. The second architecture involves saturable, receptor-occupancy pharmacology, in which an antibody or ligand administered to achieve full target occupancy elicits a maximal effect that does not increase with further increments of protein; thus, any output above the saturating level produces an identical pharmacodynamic outcome. The plateau occurs because the pharmacodynamic effect is determined by the fraction of target receptors bound, not by the free concentration of the agent: once virtually all available receptors are occupied, additional protein finds no unoccupied targets to engage, resulting in a flattened response curve, with variability above the saturating concentration being pharmacodynamically inert. The third architecture pertains to integrative immunological priming, in which the adaptive immune system samples antigen across a broad concentration spectrum and elicits a response of a similar qualitative nature, meaning that the absolute quantity of antigen generated needs only to fall within a broad range. The fourth involves self-limiting or feedback-regulated output, in which a downstream physiological control loop constrains the effect regardless of the amount of protein produced or in which the protein is consumed during its functional activity. The fifth architecture concerns binary, all-or-none molecular events, exemplified by genome editing: the genomic alteration is permanent and discrete, thereby influencing the probability and rate of editing but not the final form of the alteration; furthermore, once sufficient editors are produced to complete editing, additional amounts are immaterial except insofar as they extend the off-target window.

Restating the thesis in its bounded and defensible form: mRNA is not a general-purpose protein-manufacturing platform that happens to have some failures but a platform whose intrinsic output variability confines it, on current evidence and absent validated output control, to the threshold-response class. The correct first question about any candidate indication is therefore not whether mRNA can express the relevant protein but which class the indication belongs to. When it is a threshold response, mRNA can be excellent and further improved. Where it is exposure-controlled, delivery or sequence optimization alone will not make mRNA a safe choice, because the residual variability is structural and lies downstream of those levers; only a demonstrated post-delivery output-control mechanism would change this assessment (Figure 2 and Figure 3).

### 4.3. Implications for the mRNA 2.0 Vision

This reframing constitutes a respectful yet substantive correction to the expansive vision of an mRNA 2.0 era. That vision correctly acknowledges that the molecular and delivery toolbox has matured and that the modality is advancing beyond vaccines. However, it is incomplete and potentially overreaching to suggest that the principal remaining obstacle is delivery and that resolving this will enable the modality to address a broad spectrum of rare and common diseases. Delivery is indeed necessary but not sufficient. Even with perfect organ-targeted, immune-silent delivery, the amplification step remains uncontrolled; therefore, the modality will remain unsuitable for titratable, narrow-index drugs and will remain aligned with threshold, saturable, integrative, self-limiting, and binary pharmacology. A rigorous approach for the field, therefore, involves not pursuing every indication accessible to a protein but rather triaging indications based on their dose–response architecture, focusing on those that are inherently forgiving, while either avoiding the others or equipping the modality with closed-loop output control prior to attempting them. The remainder of this review explicitly adopts this filtering criterion, examining each therapeutic class not only to determine whether mRNA can express the relevant protein but also to assess whether the indication’s pharmacology tolerates the variability inherent in expression, thereby illustrating how genuinely suitable applications can be optimized for greater efficiency.

### 4.4. Engineering Toward Output Control and Its Limits

Presenting the constraint without acknowledging the engineering responses to it would be incomplete, although it is candidly acknowledged that such responses mitigate rather than eliminate the problem. Several approaches serve to narrow the output distribution. For instance, tightening the construct itself through high-fidelity capping, optimized untranslated regions, and codon and nucleoside choices that sustain high translation and low innate sensing effectively reduce one component of variance; however, they do not address the delivery and escape components. Cell-type-restricted expression, achieved via microRNA-responsive elements that suppress translation when a tissue-specific microRNA is prevalent, diminishes off-target effects and consequently narrows the effective therapeutic distribution by eliminating tails caused by misexpressed cells. Self-limiting circuit designs, in which the expressed protein or associated element feeds back to limit further translation, can theoretically establish a ceiling that transforms uncontrolled output into saturating output, thereby engineering a saturable architecture lacking native pharmacology. Frequent low-dose regimens combined with pharmacodynamic monitoring, akin to titrated biologics, can compensate for variability between administrations when a measurable biomarker is available, albeit at the expense of the convenience that initially rendered the modality attractive. None of these strategies confers the dose precision characteristic of small molecules; the honest conclusion remains that while engineering can slightly expand the range of manageable indications, it cannot convert a genuinely narrow-index, titratable drug into an mRNA-tractable classification. The fundamental structural fact persists: the active agent is synthesized within the patient through a process that cannot be accurately metered.

## 5. The Four Frontiers That Define mRNA 2.0

If the molecular cargo and the LNP constitute the shared substrate, then four specific capabilities distinguish a therapeutic-grade construct from a vaccine-grade one. These frontiers recur in every clinical example discussed subsequently, and preemptive identification of them elucidates the engineering rationale (Table 1).

**Table 1:** The systematic inversion from mRNA 1.0 to mRNA 2.0.

Attribute	mRNA 1.0, Vaccines	mRNA 2.0, Therapeutics
Primary purpose	Provoke adaptive immunity against an antigen	Produce a functional protein, antibody, editor or chimeric receptor
Innate immune reactivity	Desirable; acts as a built-in adjuvant	Must be silenced; inflammation is toxic and blocks translation
Dosing	Prime plus a few boosters	Lifelong re-dosing (replacement) or a single dose (editing)
Route	Local, typically intramuscular	Intravenous, inhaled, intracardiac or oral, per target organ
Target tissue	Injection-site depot and draining lymph node	Defined organ: liver, heart, lung, muscle, marrow or immune cells
Expression duration	Transient burst is sufficient	Tuned to indication: extended or deliberately brief
RNA purity bar	Tolerant of residual dsRNA (some adjuvant benefit)	Ultra-pure; dsRNA removed to preserve immune silence
LNP design goal	Efficient antigen-presenting-cell uptake	Immune-silent, organ-targeted, re-dosable delivery
RNA conformation	Linear modified mRNA	Linear, circular or self-amplifying, chosen by use
Success metric	Seroconversion and protection	Serum protein level, target engagement, edit efficiency
Representative products	Approved: tozinameran (Comirnaty), elasomeran (Spikevax), mRNA-1345 (mRESVIA), zapomeran (Kostaive)	Late stage: nexiguran ziclumeran (NTLA-2001, Phase 3), intismeran autogene (mRNA-4157, Phase 3), Descartes-08 (Phase 3), ARCT-810 (Phase 2), mRNA-3927 (Phase 1/2); no non-vaccine mRNA product is yet approved

Although a vaccine and a therapeutic share the same five-part cargo and lipid nanoparticle, nearly every design parameter is tuned in opposite directions. The mRNA 2.0 column above describes the immune-silent branch of the therapeutic paradigm, comprising protein and antibody replacement and genome editing, in which inflammation is undesirable. mRNA 2.0 also includes a second, programmable immunotherapy branch, exemplified by personalized cancer vaccines, which deliberately exploits immune activation and is discussed as a hybrid class in the cancer immunotherapy section. The immune-silence attribute in this table applies to the first branch and not to the programmable immunotherapy branch (Table 2).

**Table 2:** Messenger RNA products approved by major regulatory agencies, as of July 2026.

Product (INN and Brand)	Developer	Indication	Construct and Carrier	Regulatory Status
Tozinameran (Comirnaty)	BioNTech and Pfizer	COVID-19 prophylaxis	Linear N1-methylpseudouridine mRNA in LNP	Emergency authorizations from December 2020; full approvals from 2021 (FDA, EMA, MHRA and others) [3,5]
Elasomeran (Spikevax)	Moderna	COVID-19 prophylaxis	Linear N1-methylpseudouridine mRNA in LNP	Emergency authorizations from December 2020; full approvals from 2022 (FDA, EMA and others) [3,4]
mRNA-1345 (mRESVIA)	Moderna	Respiratory syncytial virus disease in older adults	Linear modified mRNA in LNP	FDA approval May 2024; EMA authorization 2024
Zapomeran (Kostaive)	Arcturus and CSL	COVID-19 prophylaxis	Self-amplifying RNA in LNP	Japan 2023; European Union 2025

Every approved mRNA product to date is a prophylactic vaccine. No non-vaccine mRNA therapeutic has yet received marketing authorization from a major agency. This pattern is consistent with the argument developed in this review: the indications that have reached approval are those whose pharmacology integrates antigen exposure over a wide range and therefore tolerates variable output, whereas the exposure-controlled indications remain unapproached. LNP, lipid nanoparticle.

### 5.1. Complete Immune Silence and Repeat Dosing

The initial frontier presents the greatest challenge. A non-vaccine mRNA must be meticulously designed to remain virtually undetectable by the innate immune system, owing to the toxic effects of innate activation during chronic exposure and its capacity to inhibit the translation of the therapeutic protein. Achieving this entails engineering the chemistry of the ionizable lipid, including its head group, linker, and side chains, as well as optimizing the polyethylene glycol (PEG) composition and cholesterol content to mitigate immunogenicity [66,67,74]. Additionally, ultra-purification of the mRNA is essential to eliminate double-stranded RNA contaminants, which serve as potent immunostimulants [75,76,77,78]. The objective is to develop a fully immune-silent mRNA-vehicle formulation capable of repeated administration without inducing inflammation, a requirement probably necessary for all non-vaccine applications, as identified by the framing review [6].

### 5.2. Delivery Beyond the Liver

The second frontier involves extrahepatic delivery (Figure 4). Given that most non-vaccine mRNA trials to date have focused on the liver, expanding this modality requires engineering lipid nanoparticles (LNPs) that evade hepatic uptake and target other tissues. Multiple complementary strategies are currently under investigation, including the utilization of selective organ-targeting (SORT) lipids that redirect particles to the spleen or lung through the addition of charged helper lipids [79,80]; ligand- and antibody-conjugated LNPs that bind to receptors on specific target cell types [68,81]; reductions in LNP cholesterol content to decrease hepatic uptake; and the employment of microRNA binding sites as molecular switches to restrict expression to specific cell types [82,83,84]. The frequently invoked analogy is the asialoglycoprotein receptor, which N-acetylgalactosamine (GalNAc) ligands engage to facilitate hepatic uptake of oligonucleotides; the aim is to identify equivalent molecular homing signals for tissues beyond the liver [85,86].

### 5.3. Expression-Duration Control

The third frontier involves aligning the duration of protein expression with the specific indication (Figure 5). The transient nature of expression from linear mRNA, typically around 72 h, presents a challenge for protein-replacement therapy, which necessitates sustained output. Conversely, this transient expression is advantageous for one-time genome editing, where a brief pulse of editor protein minimizes the risk of off-target effects. The repertoire of methods to extend expression duration includes circular RNAs (circRNAs), whose covalently closed structure resists exonucleases and prolongs translation [87,88]; self-amplifying RNAs (saRNAs), which encode a replicase to amplify the message, thereby requiring lower doses while achieving longer-lasting effects [20,89]; and multi-tailed or chemically modified poly(A) architectures that can augment cellular mRNA levels up to twentyfold, with protein production extending up to fourteen days [47,90]. The fundamental principle is to select the RNA conformation based on the specific indication, rather than applying a uniform conformation across all applications (Figure 5).

### 5.4. The Conformational Toolbox for Duration

Since duration is now recognized as a variable in the design process, it is appropriate to outline the three principal conformational strategies, each offering distinct advantages and disadvantages. Circular RNA is produced through the covalent ligation of its termini, thereby removing free ends that are susceptible to exonuclease activity, thus enhancing stability and extending translation duration compared to linear mRNA [87,88]. The first circular RNA therapy, RXRG001, encoding the water-channel protein aquaporin-1 for radiation-induced xerostomia, commenced dosing its initial patient in a first-in-human Phase I/IIa trial in March 2025, exemplifying the progression of this conformation from a theoretical concept to a clinical application [91]. Self-amplifying RNA originating from alphavirus genomes encodes a replicase complex consisting of four non-structural proteins along with the target gene; once inside the cell, the replicase amplifies the template, resulting in significantly higher protein levels from a reduced dose and prolonging expression duration [20,89]. Its limitations include its considerable size, which complicates packaging and delivery; its susceptibility to nuclease degradation; and its capacity to provoke innate immune responses via the double-stranded intermediates generated during replication. An existing self-amplifying construct serves as the basis for a currently approved COVID-19 vaccine, thereby demonstrating its clinical viability. The third approach, poly(A)-tail engineering, involves modifying the tail through branched, multi-tailed, or chemically capped architectures; rodent studies indicate that incorporating multiple tails can increase cellular mRNA levels by up to 20-fold, with protein expression persisting for up to 14 days. Additionally, employing multi-tailed designs in a CRISPR system has resulted in effective gene editing in murine models [47,90].

The fundamental principle is that the selection among linear, circular, and self-amplifying RNA, as well as the engineering of the tail, is dictated by the specific application. A genome editor favors the shortest effective pulse; enzyme-replacement therapy aims for the longest sustainable expression at the lowest dosing frequency; and a secreted paracrine factor may require an intermediate approach. Translational-control elements provide an additional regulatory layer: microRNA-binding sites within untranslated regions function as molecular switches, suppressing expression in cell types with abundant microRNA levels, thereby improving tissue specificity in both linear and circular systems [82,83,84] (Table 3).

### 5.5. Manufacturing, Analytics, and the Cost-of-Goods Frontier

Behind the four scientific frontiers lies a manufacturing reality that is integral to the transition to Industry 4.0. An mRNA pharmaceutical is generated by in vitro transcription from a linearized DNA template, which is subsequently capped (either cotranscriptionally or enzymatically), tailed, and purified to eliminate double-stranded RNA, uncapped species, abortive transcripts, residual template, and process enzymes. Acceptable limits for residual impurities are product-specific and are determined by dose, route, frequency, process capability, and formal risk assessment, rather than by a universal vaccine-versus-therapeutic rule. In practice, however, a repeatedly dosed systemic therapeutic tends to impose a more demanding purification burden than a few-dose intramuscular vaccine, because cumulative exposure and the need for immune silence leave less headroom for immunostimulatory contaminants such as double-stranded RNA. Meeting this burden relies on methods such as high-performance liquid chromatography, cellulose-based and chaotrope-assisted purification, and engineered polymerases that minimize byproduct formation; for such products, these tend to be treated as essential rather than optional [75,78,92,93]. The analytical evaluation of capping efficiency, molecular integrity, double-stranded RNA content, and poly(A) tail length distribution is correspondingly more rigorous, as each of these attributes directly correlates with clinical risk.

The cost of goods presents a distinctive challenge for two contrasting reasons. Regarding personalized cancer vaccines, the product is custom-manufactured for each patient; therefore, the speed and unit cost of producing a tailored plasmid template and the associated mRNA are crucial factors in determining feasibility. Enhancing the efficiency of generating personalized DNA templates is recognized as a vital element in this process [6]. Conversely, for chronic replacement therapies, patients may require numerous doses annually throughout their lives, leading to the accumulation of even modest per-dose costs into a significant barrier. In this scenario, economic factors favor manufacturing methods that offer the lowest cost per immune-compatible, highly pure dose. The advancement of continuous, intensified production processes, combined with the use of improved reagents and lyophilized, room-temperature-stable formulations, substantially contributes to making this modality viable outside cold-chain-dependent environments where the initial vaccines were administered [94].

Stability of mRNA medicines. The chemical and physical stability of the drug product is a defining practical constraint for this modality. mRNA is susceptible to hydrolysis and to ribonuclease-mediated degradation, and the encapsulating LNP is subject to lipid oxidation, ionizable-lipid degradation, particle aggregation, and cargo leakage on storage. The first-generation COVID-19 products required deep cold-chain storage, whereas therapeutic development has prioritized formulations that tolerate refrigerated or ambient conditions. Stabilization strategies include buffer and pH optimization, exclusion of divalent cations and oxidative catalysts, tromethamine and antioxidant excipients, lyophilization and spray-drying to a room-temperature-stable solid, and sequence- and tail-level design that raise intrinsic transcript stability. Stability-indicating analytics, including mRNA integrity, capping efficiency, encapsulation efficiency, particle size, and double-stranded RNA content, define shelf life and are more demanding for a repeatedly dosed systemic therapeutic than for a few-dose vaccine because cumulative exposure narrows the tolerance for degradation products.

### 5.6. Artificial Intelligence as a Cross-Cutting Accelerant

Artificial intelligence constitutes an emerging, interdisciplinary accelerant that impacts all four frontiers; however, most of the identified tools are still in the early stages of development. In the domain of cargo, generative models are beginning to surpass empirical optimization of codons, untranslated regions, and entire constructs, demonstrating significant improvements in both expression and stability, as documented by Li et al. [40], Barazandeh et al. [23], and Zhang et al. [41]. Regarding delivery, machine learning platforms are utilized to predict the transfection efficacy of potential ionizable lipids and to optimize large virtual libraries into manageable subsets for testing. Furthermore, AI-guided design is increasingly focused on creating particles with enhanced potency and biodegradability, as evidenced by research from Xu et al. [95], Han et al. [63], and Wang et al. [64]. The practical importance in the 2.0 era primarily manifests as increased speed, transforming the traditionally slow, empirical design process into a computational endeavor. It is essential to underscore that none of these advancements affect the dose-control constraint; although faster, more efficient construct design reduces certain sources of variability, the amplification step itself remains unaffected.

It is worth stating plainly how far these tools have traveled toward patients, because the distance is often overstated. The clearest clinical translation to date comes from algorithmic sequence optimization rather than generative design: LinearDesign, which jointly optimizes codon usage and secondary-structure stability, produced COVID-19 vaccine constructs whose improved stability and immunogenicity in preclinical comparisons supported advancing algorithmically optimized candidates into clinical evaluation. Deep-learning-optimized untranslated regions and machine-learning-selected ionizable lipids, including candidates identified through platforms such as AGILE, have generated constructs and carriers that outperform empirically designed comparators in preclinical models, and several developers now describe computationally optimized sequences within clinical-stage pipelines. What has not yet occurred is the registration of any mRNA therapeutic whose sequence or carrier was wholly machine-generated and approved on that basis. The accurate summary is therefore that artificial intelligence is already shaping clinical-stage constructs at the sequence and lipid selection levels, while fully generative design remains predominantly preclinical.

## 6. Secreted Protein Therapeutics: The First Step Beyond Vaccines

### 6.1. VEGFA in Heart Disease

The initial human trial of an mRNA therapeutic beyond vaccines involved testing an mRNA encoding vascular endothelial growth factor A (VEGFA) to stimulate regenerative angiogenesis, building upon five years of preclinical research conducted in both small and large animal models [96,97,98]. In patients with type 2 diabetes, intradermal administration of VEGFA mRNA resulted in a dose-dependent increase in local protein levels and temporarily reversed vascular dysfunction without inducing adverse effects [99]. Extending this methodology to cardiac applications encountered a significant obstacle: the first-generation lipid nanoparticles (LNPs) proved cardiotoxic. The solution was an unexpected discovery that naked, chemically modified VEGFA mRNA could be directly taken up by cardiomyocytes following intracardiac injection, thus enabling localized, therapeutic secretion levels [100]. The candidate entity, AZD8601, was evaluated in a small Phase II study involving patients undergoing coronary artery bypass grafting. The trial achieved its safety and tolerability endpoints; although it was not powered for efficacy, some indications of benefit emerged after more than 25 intramyocardial injections per patient [101].

The VEGFA program exemplifies the transition from version 1.0 to 2.0 and serves as an illustrative case within the field. It demonstrated that messenger RNA (mRNA) can produce a secreted paracrine factor, for which transient expression by a subset of cells is sufficient. This finding paves the way for the application of secreted proteins in various indications. It supports cardiovascular repair and localized angiogenesis [102], while related VEGF-delivery work supports renal recovery [103] and combined BMP-2 and VEGF-A modified mRNA supports bone repair [104], with wound healing and tissue grafting as adjacent opportunities. Additionally, the program revealed three critical requirements for therapeutic-grade applications that vaccines do not typically face: first, the expression was confined to cells immediately adjacent to the needle track, highlighting the necessity for efficient systemic delivery; second, the cardiotoxicity associated with first-generation lipid nanoparticle (LNP) formulations underscored the need for immune-silent delivery vehicles; and third, the transient nature of expression emphasized the importance of controlling duration.

Upon reviewing the dose–response filter, local regenerative angiogenesis emerges as a relatively forgiving target, which partly explains its selection as a first-line approach. The therapeutic goal is to surpass a local threshold of angiogenic signaling adequate to initiate vessel growth. The subsequent biological cascade is inherently regulated and self-limiting rather than a straightforward linear function of VEGFA concentration. Consequently, a multiplicative uncertainty in local protein production translates into a significantly smaller uncertainty in the vascular response. The primary risk associated with excess VEGFA, namely, the development of disorganized or leaky vessels, imposes an upper limit that necessitates some degree of control. However, the therapeutic window remains broad rather than narrow. This characteristic is well-suited to the capabilities of mRNA technology. Conversely, employing the same secreted-protein strategy to deliver a hormone characterized by a steep, systemic, and narrow-dose–response curve, where both insufficient and modestly excessive levels could cause clinical harm, would be unsuitable within the current framework, irrespective of the construct’s sophistication.

### 6.2. IL-2 Mutants in Autoimmune Diseases

Interleukin-2 (IL-2) is essential for the differentiation of regulatory T cells (Treg) and the maintenance of immune homeostasis. Impairments in IL-2 signaling have been associated with various autoimmune disorders [105,106]. While low-dose IL-2 selectively activates Treg cells, higher doses may also stimulate effector and natural killer cells, resulting in varied clinical outcomes [107,108]. An mRNA-based strategy to address these challenges involves mRNA-6231, an LNP-encapsulated mRNA encoding a Treg-selective IL-2 mutein fused to human serum albumin to extend its half-life. This approach has demonstrated the ability to selectively expand Treg cells and diminish disease severity in preclinical models [109,110]. A semi-mechanistic kinetic-pharmacodynamic model developed in non-human primates informed initial dosing in humans; however, Moderna discontinued further development based on undisclosed trial data [6]. This instance highlights both the potential of engineering a targeted immunomodulator and the difficulties in translating a narrow therapeutic window observed in primates to human patients.

The IL-2 case functions as a cautionary parallel to VEGFA within the same secreted-protein category, exemplifying the filter’s precise function. IL-2 pharmacology is inherently unforgiving: the entire therapeutic strategy depends on maintaining a narrow concentration range wherein Treg cells are selectively stimulated. Elevated levels beyond this range recruit effector and natural killer cells, thereby reversing the intended therapeutic effect. This represents a steep, bidirectional, narrow-index dose–response curve, an architecture that inadequately accommodates variability in mRNA output. Modifications aimed at extending half-life and meticulous kinetic-pharmacodynamic modeling were efforts to regulate an output that the modality inherently cannot precisely control. Because the reason for discontinuation was not disclosed, it cannot be treated as evidence that the present taxonomy predicted the outcome. It is offered here only as an illustration of the development uncertainty that accompanies narrow-window immunomodulators, whose steep bidirectional pharmacology is independently difficult to reconcile with variable mRNA output. The key inference is not that mRNA cannot produce IL-2, but rather that the pharmacological characteristics of the indication, rather than the protein’s identity, determine its suitability.

## 7. mRNA-Encoded Antibodies

A natural progression of secreted-protein therapy involves enabling patients to produce antibodies in vivo, thereby eliminating the need for manufacturing, purification, and logistical transportation of recombinant proteins while concurrently reducing the time from initial design to clinical application [111,112]. The identical LNP-mRNA payload can encode either agonistic or antagonistic, monospecific or bispecific antibodies. In oncology, BNT142 is an intravenous LNP-mRNA formulation encoding a bispecific antibody that targets Claudin-6 (CLDN6) on tumor cells and CD3 on T cells. Upon hepatic uptake, the patient produces the engineered antibody, which is detectable in serum, reaches its peak concentration between 24 and 72 h post-dosing, and induces dose-dependent cytokine elevations alongside early indicators of activity in CLDN6-positive solid tumors, including ovarian cancer [113,114].

Concerning infectious diseases, mRNA-1944 encodes a human monoclonal antibody (CHKV-24 IgG) targeting the E2 glycoprotein of the Chikungunya virus. It has demonstrated dose-dependent antibody levels, which are predicted to be protective, in a Phase 1 clinical trial [115]. The program also underscored a cross-species translational risk relevant to all systemic mRNA therapeutics: while non-human primates tolerated high doses, humans receiving one-tenth the preclinical dose experienced frequent treatment-related adverse events, leading to the candidate’s inability to advance. This contrast with vaccination is instructive. A vaccine aims to provoke an immune response; by contrast, an mRNA-encoded antibody is intended to make the patient function as a stable bioreactor for a specific protein drug, with success evaluated by serum concentration and target engagement rather than seroconversion.

When evaluated through the lens of the dose–response relationship, antibody pharmacology is predominantly favorable, underpinning its consideration as a promising class. Therapeutic antibodies are typically administered at doses aimed at saturating their respective targets. Once target occupancy is achieved, the pharmacodynamic response reaches a plateau, rendering any output exceeding the saturating threshold largely inconsequential, as the inherent variability in this modality is absorbed. A bispecific T cell engager, such as the Claudin-6 candidate, is comparatively less accommodating because excessively high concentrations of the engager can induce excessive cytokine release. This phenomenon establishes a ceiling on tolerable output and effectively narrows the operational window from above; the observed dose-dependent increases in cytokine levels exemplify the visible boundary of this ceiling. The practical implication of these observations is that mRNA-encoded antibodies are particularly well suited for targets that are saturable and where the safety margin is substantial. Conversely, such modalities necessitate output regulation or precise dose fractionation when, as with potent T cell engagers, the upper limit of tolerability nears the effective dose. The experience with Chikungunya, which demonstrated a narrow tolerable margin in humans, further indicates that the suitability of these approaches is more influenced by the indication and the potency of the molecule than by the feasibility of expression.

## 8. Enzyme Replacement Therapy for Rare Diseases

Recombinant enzyme replacement therapies were among the initial treatments devised for numerous single-protein-deficiency disorders; however, their effectiveness is limited by their inability to access the appropriate intracellular compartments or tissues [116,117]. Adeno-associated virus (AAV) gene therapy provides sustained expression from a single administration but is subject to significant limitations: immune responses targeting the capsid, neutralizing antibodies that hinder re-administration, a transgene capacity limit of approximately five kilobases, and the dilution of episomal vectors due to hepatocyte turnover, which is particularly problematic in pediatric patients whose livers are still developing [118,119]. mRNA-based replacement therapy presents an attractive alternative, especially in contexts where these constraints are most impactful, contingent upon the consistent manufacture of high-quality, ultra-pure RNA suitable for repeated dosing. Furthermore, it offers two notable capabilities: concurrent delivery of multiple proteins and mitochondrial targeting, features that are challenging to achieve with both recombinant proteins and AAV [6].

Enzyme replacement therapy, when assessed through the dose–response framework, emerges as the most beneficial modality for mRNA. It is important to clarify the mechanistic rationale behind this, as it has broad applicability. Many metabolic enzyme deficiencies are diseases characterized by a lack of catalytic activity, where patients do not produce sufficient enzymes to effectively regulate an accumulating substrate. Restoring even a modest fraction of normal enzymatic activity, on the order of ten to twenty percent in several lysosomal and metabolic disorders, may substantially reduce substrate accumulation and attenuate phenotypic manifestations. The correcting threshold and the degree of tissue correction are disease-, tissue-, and endpoint-specific: they differ across Fabry, Gaucher, Pompe, and related disorders, and established structural or organ pathology can persist despite partial restoration of enzyme activity. When the enzyme operates well below saturation on an abundant substrate, additional activity beyond the effective threshold is metabolically silent. This applies only when excess activity is benign; it does not apply to enzymes where overactivity, expression in an inappropriate tissue or compartment, immunogenicity of the expressed protein, or depletion of a necessary substrate could cause harm. Such targets should be regarded as exposure-controlled rather than threshold-response. As a result, the dose–response relationship adopts a stepped profile with a significantly prolonged flat plateau: below the threshold, disease manifests; above it, health persists across a broad spectrum of expression levels. This structural configuration inherently tolerates the variability in mRNA expression, as such variability mainly occurs within the flat region, where it has no clinical impact. Therefore, the therapeutic goal simplifies to reliably surpassing the threshold rather than attaining a precise enzymatic level, which mRNA may achieve if delivery, expression duration, tissue localization, and immunogenicity are properly managed. This logical framework also explains why these therapeutic indications accommodate dose and patient variability, which would typically exclude a drug with a narrow therapeutic window.

### 8.1. Propionic and Methylmalonic Acidemia

Propionic acidemia results from a deficiency of the intramitochondrial enzyme propionyl-coenzyme A carboxylase (PCC), which traditionally facilitates the breakdown of branched-chain amino acids. In the absence of this enzyme, toxic metabolites accumulate, leading to potentially life-threatening metabolic decompensation events. A pioneering phase 1/2 trial involved the intravenous infusion of an LNP-mRNA encoding the separate alpha and beta subunits of PCC, resulting in a 70 percent reduction in the risk of decompensation events among a subset of patients [120]. This achievement is significant not only from a clinical perspective but also mechanistically, as it necessitated the expression of two distinct proteins within a single cell, their correct routing to the mitochondrion, and the attainment of therapeutic levels, an accomplishment beyond the capabilities of a vaccine-grade construct.

Methylmalonic acidemia, which results from a deficiency of mitochondrial methylmalonyl-CoA mutase (MMUT), follows a similar rationale [121,122]. The initial candidate, mRNA-3704, was discontinued amid the COVID-19 pandemic; subsequent development led to the second-generation mRNA-3705, which was re-engineered to optimize expression and mitochondrial localization, reduce expression in antigen-presenting cells, and incorporate N1-methylpseudouridine along with mitochondrial targeting sequences. This re-engineering resulted in greater and more sustained reductions in plasma methylmalonic acid in murine models [123]. As recorded on ClinicalTrials.gov (accessed 23 July 2026), the global Phase 1/2 dose-optimization study (NCT04899310) is listed as terminated, whereas its long-term open-label extension (NCT05295433) is listed as recruiting [124,125]. The iterative process of re-engineering across successive generations exemplifies the 2.0 mindset, characterized by the continual retuning of cargo to achieve performance specific to the cellular compartment.

### 8.2. Glycogen Storage Disease and the Three-Modality Comparison

Glycogen storage disease type 1a (GSD1a), caused by a deficiency of glucose-6-phosphatase (G6Pase), presents a noteworthy case as three modalities are being evaluated concurrently [126,127]. Ultragenyx’s DTX401, an AAV8 vector expressing G6Pase, achieved its primary endpoint in a phase 3 clinical trial [128]. Moderna’s mRNA-3745, an LNP-mRNA encoding G6Pase, demonstrated the ability to restore enzyme activity and normalize fasting glucose levels in preclinical studies; however, at its Analyst Day on 20 November 2025, Moderna announced that the GSD1a program would not advance to Phase 2, despite preclinical evidence indicating it was well tolerated across repeated dosing [129,130,131]. Beam’s BEAM-301 employs an mRNA-encoded adenine base editor with a guide RNA to directly correct the G6Pase R83C mutation, successfully correcting up to 60 percent of the variant in hepatocytes in rodent models [132]. This comparative analysis elucidates where mRNA replacement therapy might possess an advantage, particularly in pediatric applications, where AAV vectors are less effective due to hepatocyte turnover and where pre-existing anti-capsid antibodies can impede delivery [133,134].

### 8.3. Ornithine Transcarbamylase Deficiency and the Purity Lesson

Numerous mRNA therapeutic interventions have been explored for the treatment of ornithine transcarbamylase (OTC) deficiency, a condition characterized by elevated blood ammonia levels. Translate Bio’s MRT5201 was discontinued in 2019 due to safety concerns and pharmacokinetic challenges, which the company attributed to suboptimal LNP features. This case exemplifies the importance of next-generation, immune-silent, ultra-pure formulations in this domain [117]. Arcturus’s ARCT-810, delivered utilizing its LUNAR lipid nanoparticle (LNP) technology, completed a Phase I clinical trial, and on 30 June 2025, Arcturus reported positive interim Phase 2 multiple-dose data together with an ongoing U.S. Phase 2 open-label study [135]. The development experience associated with OTC underscores the significance of purity and immune silence as fundamental prerequisites rather than mere incremental improvements.

Throughout the examples of enzyme replacement therapy, two key principles remain consistent. The pharmacology demonstrates leniency because correcting a catalytic deficiency beyond a certain threshold allows for significant variation in expression levels, which explains why this category is the natural domain for mRNA applications. However, the persistent challenge lies in the delivery vehicle, as first-generation lipid nanoparticles (LNPs) were not designed for repeated, systemic, immune-silent administration. Additionally, recent safety signals regarding base editors underscore the need for next-generation LNPs specifically optimized for secure hepatic uptake. An important exception within this category concerns enzymes whose excess can be detrimental; in such cases, the upper limits of expression reassert themselves, and the tolerant architecture no longer applies. Targets of this nature necessitate the implementation of the output control mechanisms previously discussed and should be approached with caution.

## 9. Personalized Cancer Immunotherapy

Therapeutic cancer vaccines occupy an intermediate position between 1.0 and 2.0: they still aim to provoke immunity, but they exploit the speed and programmability that define the new era. The concept is to identify the neoantigens unique to a patient’s tumor, encode several dozen of them in mRNA, and deliver them to antigen-presenting cells, which present the epitopes and prime a T cell response against the cancer [136,137]. Speed is essential because the vaccine must be manufactured for each patient, and this is a natural fit for the rapid, multiplexed mRNA platform.

The leading candidate, intismeran autogene (mRNA-4157), encodes up to 34 patient-specific neoantigens and, in combination with the checkpoint inhibitor pembrolizumab, has demonstrated a reduction in recurrence in resected melanoma in a randomized phase 2b trial [138]. On 20 January 2026, Moderna and Merck announced that, based on a median five-year follow-up of the Phase 2b KEYNOTE-942 study, the combination therapy reduced the risk of recurrence or death by 49% compared with pembrolizumab alone. Additionally, eight Phase 2 and Phase 3 clinical trials are currently in progress across melanoma and other tumor types [139]. A significant question is whether this approach can be generalized to tumors with low mutational burdens. Pancreatic ductal adenocarcinoma (PDAC) presents a substantial challenge due to its low neoantigen load, which hampers the elicitation of tumor-specific T cell responses [140]. Autogene cevumeran, an individualized mRNA-lipoplex vaccine encoding up to 20 neoantigens, when combined with surgery, atezolizumab, and chemotherapy, induced durable, functional T cells that persisted for up to four years and delayed recurrence in responders [141,142]. An alternative to fully personalized vaccines involves off-the-shelf mRNA vaccines targeting shared antigens conserved across tumors, such as BNT111 in melanoma and BNT116 in non-small-cell lung cancer [6].

The cancer-vaccine programs demonstrate that the rapidity and programmability of the platform, rather than its immunostimulatory capacity alone, constitute the primary advancement of version 2.0. A personalized vaccine requires sequencing a patient’s tumor, computationally predicting the neoantigens most likely to elicit an effective T cell response, and manufacturing an mRNA encoding multiple neoantigens, all within a clinically relevant timeframe following surgery. None of these processes are achievable with a conventional protein vaccine tailored to individual patients; it is the rapid, multiplexed, sequence-defined nature of mRNA that makes individualized therapy possible. Furthermore, the delivery formulation differs from that of systemic therapeutics: lipoplex and lipid nanoparticle (LNP) formulations are optimized to target antigen-presenting cells within lymphoid tissues, unlike the immune-silent, organ-specific targeting aim of replacement therapies. In this context, cancer immunotherapy embodies a hybrid approach, retaining the immune-activating purpose of version 1.0 while fully utilizing the design speed and precision of version 2.0. Consequently, it stands as one of the first non-infectious indications to demonstrate robust, randomized, positive clinical data.

Concerning the dose–response filter, therapeutic cancer vaccines are comfortably categorized within the forgiving group, analogous to prophylactic vaccines. The indicator is an integrated immunological response rather than protein concentration. The adaptive immune system appraises antigens produced across a broad spectrum and triggers a qualitatively comparable priming response. Consequently, the specific quantity of each translated neoantigen need only be within a broad range rather than at a precise value. Variability in mRNA output, therefore, is largely inconsequential in this context, which substantially explains why cancer immunotherapy, akin to vaccination, remains among the most effective current applications of this modality. The genuine challenges in this domain are immunological and computational, particularly in selecting neoantigens that stimulate functional T cells and in addressing low mutational burdens in tumors such as pancreatic cancer, rather than in attaining pharmacokinetic precision. This exemplifies the broader principle that mRNA technology performs optimally where biological systems can accommodate and tolerate variability and encounters difficulties when precise dosing is requisite.

## 10. mRNA-Based Genome Modification: The One-And-Done Logic

In the context of genome editing, the transient nature that impairs replacement therapy becomes advantageous. A brief exposure to the editor suffices to induce a permanent modification, and restricting the duration of editor expression minimizes off-target effects. This benefit forms the foundation of a new generation of mRNA-based gene, base, and prime editors [143,144].

The reasoning warrants emphasis because it inverts the typical correlation between a drug’s pharmacokinetics and its effect. In nearly all other therapeutic categories, prolonged exposure is advantageous; however, for a genome editor, the genetic alteration is permanent regardless of the editor’s persistence. Consequently, the optimal approach is the shortest exposure that ensures complete editing. A protein that remains in the system merely increases the likelihood of the editor acting at unintended sites. mRNA is nearly ideal for this purpose: it generates a transient, self-limiting burst of editor protein and is subsequently degraded, leaving no residual template. This sharply contrasts with viral delivery systems, which can induce prolonged expression and consequently a broader window for off-target effects. This distinction explains why LNP-mRNA has become the preferred vehicle for in vivo editing, despite the durability advantages associated with viral vectors for simple gene addition. The three primary editing architectures, nuclease, base editor, and prime editor, cover a spectrum from creating a break to disrupt a gene to modifying single bases without double-strand breaks to synthesizing new sequences from a template. mRNA can deliver all three [144,145,146].

Genome editing exemplifies a binary, all-or-none pharmacological process, making it inherently well-suited for messenger RNA (mRNA) applications despite, and indeed because of, the modality’s output variability. The therapeutic outcome involves a discrete alteration to the genome, which either occurs at a specific locus or does not. The quantity of editor protein present influences both the probability and the rate at which editing is completed across the target cell population; however, it does not modify the final edited state in a successfully modified cell. Once the desired proportion of cells has been edited, additional editing provides no further advantage and may only increase the risk of off-target effects, underscoring the advantage of transience. Variability in the amplification factor thus translates into variability in editing kinetics and the proportion of cells edited at a given dose, parameters that can be characterized and adjusted against a measurable editing endpoint, rather than into the moment-to-moment concentration fluctuations that could destabilize a narrow therapeutic window in continuous drug delivery. The challenge posed by mRNA titration for titratable proteins is largely mitigated by the binary nature of the editing endpoint. However, it is important to note that off-target effects resulting from overshoot are not benign; therefore, the objective is to achieve the minimum editor exposure necessary to attain the desired editing rate. This approach aligns safety considerations with the modality’s inherent transience.

### 10.1. Gene Disruption: The Transthyretin Landmark

The initial in vivo CRISPR gene-editing clinical trial in humans involved the administration of a lipid nanoparticle (LNP) containing messenger RNA (mRNA) encoding the Cas9 nuclease and a single-guide RNA to the livers of patients diagnosed with transthyretin amyloidosis. This disease is characterized by the secretion of misfolded transthyretin, which forms amyloid deposits in the myocardium, ultimately leading to irreversible heart failure [147]. The therapeutic intervention, designated NTLA-2001, leveraged the potential of a single administration of gene editing and the hepatic tropism of intravenously administered LNPs. Notably, it resulted in an average 87% reduction in serum transthyretin levels in the high-dose arm four weeks after a single treatment [147]. Phase 3 clinical trials targeting cardiomyopathy (MAGNITUDE) and polyneuropathy (MAGNITUDE-2) commenced in 2024 [148,149]. Both trials were placed on FDA clinical hold in October 2025 following a serious liver adverse event and a patient death; the holds were subsequently lifted, on MAGNITUDE-2 in January 2026 and on MAGNITUDE in March 2026, with enhanced hepatic safety monitoring, and enrollment resumed [150,151]. Compared to adeno-associated virus (AAV) vectors, the mRNA-based approach provides transient production of the gene editor, thereby reducing off-target risks, and imposes virtually no limitation on cargo size, in contrast to the approximately 5-kilobase capacity of AAV vectors [152,153].

### 10.2. Base Editing: PCSK9 and the Dual-Nascent-Technology Problem

Base editing rectifies individual nucleotides without inducing double-strand breaks, employing fusions of a Cas protein with a deaminase [146]. Verve Therapeutics has reported clinical proof-of-concept for VERVE-102, an LNP-mRNA that delivers an adenine base editor that introduces a stop codon in PCSK9 to achieve sustained reduction in LDL cholesterol [154,155,156]. The program also highlighted the challenge of integrating two technologies, each currently lacking full clinical validation: an earlier candidate, VERVE-101, caused a transient increase in liver enzymes and thrombocytopenia in one patient, prompting Verve to suspend its dosing in 2024 [157]. Subsequently, Verve redirected its focus to a GalNAc-modified LNP intended to enhance the safety profile [158]. Another candidate, YOLT-101, utilizing GalNAc-modified LNPs, also demonstrated a dose-dependent reduction in PCSK9 [159]. Considering that the approved and long-lasting PCSK9-lowering siRNA, inclisiran, is already available and requires only a few administrations per year [160,161], the pertinent inquiry pertains to whether a permanent genetic edit will attract interest beyond patients with adherence challenges and how infrequent off-target single-base modifications will be identified and monitored.

### 10.3. The N-of-1 Milestone

Base editing achieved a significant milestone when it was successfully employed to treat a newborn with carbamoyl-phosphate synthetase 1 (CPS1) deficiency, a severe urea cycle disorder associated with high mortality rates in early infancy, representing the world’s first N-of-1 CRISPR therapy [162,163]. Whole-genome sequencing performed at birth identified the specific mutation; subsequently, a patient-specific hepatocyte cell line and a base-editing strategy were developed. Safety evaluations in mouse and primate models supported preclinical safety evaluation, and FDA authorization followed within approximately six months. The infant received two intravenous infusions and demonstrated a reduction in blood ammonia levels and improved protein tolerance without serious adverse events [162,164]. This unprecedented acceleration, from design to clinical application, exemplifies how the integration of sequencing technology, base editing, lipid nanoparticle (LNP) delivery systems, and regulatory collaboration can significantly shorten timelines traditionally measured in years.

### 10.4. Gene Insertion and the Size Advantage

Prime editing and large-cargo insertion further exploit the absence of a strict mRNA size limit. Prime editing employs a reverse-transcriptase-nickase fusion, guide RNAs, and a template; however, the fusion protein exceeds 6 kilobases and therefore surpasses the capacity of AAV vectors [145]. LNP-mRNA delivery, with an average payload of 2.8 mRNAs per nanoparticle at a length of 1.9 kilobases, provides an alternative, with its payload capacity improving as core LNP technology advances [165]. It has been demonstrated that a single LNP can facilitate the insertion of an entire transcriptional unit into a genomic safe harbor using retrotransposon technology, without the need to employ CRISPR or guide RNAs. Furthermore, similar all-RNA integration systems utilizing clinically suitable LNPs have been reported [166,167]. Although these technologies remain primarily in the preclinical stage, and at least one developer of an integrase platform has ceased operations, the structural and durable size advantage over AAV persists [168].

### 10.5. Hematopoietic Targeting

The delivery of genome editors to hematopoietic stem cells could revolutionize the treatment of blood disorders. The 2023 approval of the first CRISPR therapy, exagamglogene autotemcel (Casgevy), for sickle cell disease and beta-thalassemia validated the underlying biology but necessitated ex vivo modification, extensive preconditioning, and autologous transplantation at a cost that is nearly prohibitive [169,170]. The ambitious 2.0 objective aims at in vivo delivery to hematopoietic stem cells, circumventing preconditioning, and is being pursued through the development of bone-homing lipid nanoparticles (LNPs), ligand- and antibody-coated particles, and barcoding-guided discovery of marrow-tropic formulations [171,172,173]. Additionally, a barcoding strategy combined with single-cell nanoparticle-targeting sequencing has identified an LNP capable of delivering mRNA to hematopoietic stem and progenitor cells in primates without a targeting moiety, with direct implications for hematological diseases.

## 11. In Vivo Immune-Cell Reprogramming

Perhaps the most notable convergence of mRNA technology with cell therapy is the in vivo generation of chimeric antigen receptor (CAR) cells. Traditional CAR-T therapy requires ex vivo isolation, viral transduction, expansion, and lymphodepleting preconditioning, procedures that are costly, time-consuming, and associated with toxicity risks. mRNA reprogramming provides a transient, non-integrating alternative with more predictable pharmacokinetics and a diminished risk of insertional mutagenesis [174,175].

The comparison with established CAR-T therapy offers valuable insights into multiple facets simultaneously. Approved CAR-T products are produced by collecting a patient’s T cells, transducing them with a virus that permanently incorporates the receptor gene, expanding these cells over several days to weeks within a specialized manufacturing facility, and subsequently reinfusing them following chemotherapy that reduces the existing lymphocyte population to create space. While the results are durable, this approach is costly, time-consuming, limited to manufacturing centers, and associated with significant toxicities, such as cytokine release syndrome and neurotoxicity, partially attributable to the persistence and proliferation of engineered cells. Conversely, an mRNA-based receptor is inherently transient: the cell expresses the chimeric receptor only as long as the mRNA remains viable, after which expression ceases. This method sacrifices durability but provides a self-limiting safety profile, eliminates the risk of insertional mutagenesis linked to integrating viruses, and in the fully in vivo variant, removes the manufacturing step altogether by generating engineered cells within the patient. The very transience that makes mRNA unsuitable for a one-time permanent cure also renders it suitable for a controllable, repeatable immunological intervention.

The most advanced example is Cartesian Therapeutics’ Descartes-08, an autologous mRNA CAR-T therapy targeting B cell maturation antigen, in which patient T cells are transfected ex vivo with CAR-encoding mRNA and returned without preconditioning. In a phase 2b trial in generalized myasthenia gravis, 83 percent of patients achieved a clinically meaningful response at month 12, with durable benefits and an acceptable safety profile [176,177]. As of mid-2026, the Phase 3 AURORA trial in generalized myasthenia gravis is enrolling (NCT06799247) [178]. A Phase 2 trial in systemic lupus erythematosus generated an encouraging initial efficacy signal in 2025, but the company announced in November 2025 that it planned to pause further Descartes-08 development in lupus, including Phase 2 enrollment, to prioritize myasthenia gravis and myositis [179]. Because the mRNA does not integrate and expression is transient, the engineered cells are self-limiting, which is expected to reduce severe toxicities such as cytokine release syndrome.

The subsequent frontier beyond ex vivo research involves the complete in vivo generation of CARs. In murine models, ligand-decorated lipid nanoparticles (LNPs) carrying CAR messenger RNA (mRNA) have been used to reprogram T cells in vivo, eliminating the need for isolation or viral transduction and thereby significantly enhancing scalability [180]. A CD5-targeted LNP delivering mRNA encoding a chimeric antigen receptor (CAR) against fibroblast activation protein produced transient anti-fibrotic CAR-T cells that mitigated cardiac fibrosis and improved cardiac function in murine models, thereby extending applications from oncology to cardiovascular disease [180]. A study conducted by Capstan Therapeutics encapsulated anti-CD19 CAR mRNA within a T cell-targeting lipid nanoparticle, resulting in the generation of engineered CD8-positive T cells within the blood, bone marrow, spleen, and lymph nodes of mice and successfully achieving tumor control in both murine and primate models [181]. These findings provide proof of concept that a patient’s own immune cells can be reprogrammed in situ, with substantial implications for both oncological and autoimmune conditions.

A related and conceptually opposing application is tolerization. Whereas a vaccine introduces an antigen accompanied by robust danger signals to elicit an immune response, a tolerizing therapy administers an antigen devoid of all danger signals to foster immune tolerance. In a murine model of multiple sclerosis, immune-silent lipid nanoparticles (LNPs) delivering messenger RNA (mRNA) encoding an autoantigen to antigen-presenting cells, in the absence of co-stimulation, resulted in antigen-specific tolerance [182,183]. This application predominantly relies on the principles of immune silence and ultra-purification, as even trace amounts of double-stranded RNA could transform a tolerogenic dose into an immunogenic one.

About dose–response considerations, in vivo cell reprogramming presents significant advantages owing to its inherently self-limiting nature in two respects. Firstly, the engineered receptor is expressed temporarily via non-integrating mRNA, which causes the reprogrammed cell to revert once the message diminishes. Secondly, the downstream effects, whether involving tumor cell eradication or modulation of autoimmune responses, are governed by cellular and immunological feedback mechanisms rather than a direct proportional relationship with receptor abundance. The number of cells reprogrammed at a given dosage may vary; however, the therapeutic response constitutes an integrated biological reaction with intrinsic regulatory processes, rather than a protein concentration that must be maintained at a specific level. Tolerization similarly functions as an integrated process, depending on the qualitative presentation of the antigen rather than on its precise amount. Consequently, both modalities can accommodate output variability, aligning with the principles of mRNA 2.0, analogous to editing and immunization. The primary requirements remain qualitative, specifically the silencing of innate signals for tolerization and the targeting of the appropriate cell subset for reprogramming, rather than precise dosing measurements.

## 12. mRNA 2.0 Among the Modalities: Convergence, Not Rivalry

Before analyzing how 2.0 accelerates delivery, it is beneficial to situate the modality within its spectrum of alternatives, as this comparison clarifies its distinctive contributions. The main competing or complementary modalities encompass recombinant proteins, antibody-based therapeutics, small interfering RNA, antisense oligonucleotides, adeno-associated virus gene therapy, and ex vivo cell therapy. Each modality has innate strengths that mRNA does not replace, as well as weaknesses that mRNA may potentially address.

Compared to recombinant proteins and antibodies, messenger RNA (mRNA) facilitates in vivo synthesis, thereby obviating the need for the production, purification, formulation, and cold-chain logistics typically associated with proteins. Additionally, it permits a swift transition between constructs through sequence editing rather than re-engineering bioprocesses [112]. Unlike small interfering RNA and antisense oligonucleotides, which operate via gene silencing, mRNA functions by conferring a new activity, expressing a protein, antibody, or editor, rendering these modalities complementary rather than interchangeable. Notably, for various hepatic targets, such as transthyretin and PCSK9, durable small interfering RNA (siRNA) already offers a reliable alternative, setting a high benchmark that a single genome-editing event must surpass [160,161]. Regarding adeno-associated virus (AAV) gene therapy, conventional mRNA yields transient and potentially repeatable expression, whereas AAV is generally used for durable, single-administration expression whose re-dosing is often constrained by anti-capsid immunity. The mRNA route circumvents strict cargo-size limitations, avoids insertional mutagenesis, and is not limited by pre-existing or treatment-induced anti-capsid immunity that can preclude AAV re-administration. Such a trade-off is advantageous in contexts such as gene editing, pediatric applications in which vector dilution diminishes AAV efficacy, and conditions requiring titratable or repeated dosing [118,119,152]. In the realm of ex vivo cell therapy, in vivo mRNA reprogramming offers the potential to eliminate the costs, delays, and preconditioning toxicity associated with ex vivo cell manufacturing [180,181].

The most precise interpretation of mRNA 2.0 is therefore as a convergence platform. The transthyretin and CPS1 initiatives concurrently involve mRNA, gene editing, and LNP delivery; the in vivo CAR programs simultaneously encompass mRNA and cell therapy; and AI-engineered sequences and lipids constitute the foundation of all these advancements. The progression of this field is characterized not by mRNA, gene therapy, or cell therapy as isolated modalities but rather by the integration of these previously separate disciplines into a unified, programmable platform enabled by computational design [6,175].

## 13. How 2.0 Enables Improved and Faster Delivery

The overarching theme across the preceding sections is that the next generation of mRNA therapeutics will be distinguished by advances in delivery mechanisms. Furthermore, improvements in delivery efficiency are simultaneously linked to increased speed. Multiple technological strategies are progressively converging towards this aim.

Firstly, artificial intelligence-guided molecular design significantly diminishes the duration required to transition from conceptualization to an optimized construct. Generative models are presently capable of designing untranslated regions (UTRs), codon-optimized coding sequences, polyadenylation (poly(A)) lengths, and capping strategies. Furthermore, platforms such as AGILE can predict the transfection efficiency of ionizable lipids across diverse cell types, thereby focusing extensive virtual libraries to a select few candidates suitable for experimental testing [95,184]. Models that concurrently optimize both the 5′ UTR and coding sequence, such as LinearDesign2, achieve a balance among translation initiation efficiency, codon adaptation, and folding stability within a unified process, as documented in a preprint [185], whereas other deep-learning methodologies specifically target 5′ UTR design [186]. The practical implication of these developments is that the traditionally empirical, time-intensive design phase is increasingly becoming computational and expeditious.

Secondly, advancements in manufacturing and formulation techniques are decreasing the time from sequence identification to product development. Since patients can be induced to produce therapeutic antibodies or enzymes in vivo, the traditionally lengthy processes of recombinant protein production, purification, and cold-chain distribution can be entirely circumvented for certain indications. In the context of personalized cancer vaccines, the primary limiting factor is the interval between tumor sequencing and formulation; reducing this interval directly expands the patient population eligible for these vaccines [142]. Furthermore, continuous freeze-drying and lyophilized formulations that remain stable at room temperature, as utilized in inhaled vaccine candidates, lessen the logistical challenges that previously restricted vaccines, such as 1.0 [94].

Thirdly, innovations in delivery methods are opening access to organs that were previously inaccessible, employing non-invasive approaches. Inhaled, nebulization-stable cationic lipid nanoparticles (LNPs) are in clinical development for rare airway diseases, with formulations stored at room temperature, like conventional inhalers. As of late 2025, Arcturus reported interim Phase 2 data for the inhaled cystic fibrosis candidate ARCT-032, and ReCode’s inhaled DNAI1 mRNA candidate RCT1100 for primary ciliary dyskinesia progressed from first-in-human Phase 1 testing to a Phase 1b study that reported initial evidence of biological activity in 2026 [187,188,189]. An engineered oral capsule, known as RNACap, protects mRNA from gastric degradation and facilitates its release in the intestine, thereby enabling self-administration for chronic intestinal diseases [190]. Additionally, SORT lipids, GalNAc ligands, antibody conjugation, and barcoding-guided discovery are expanding the range of targetable tissues [79,173,191]. Each of these advancements constitutes both a reach enhancement and a speed increase, as self-administered or organ-specific products reduce the reliance on clinical infrastructure and decrease the number of procedures required per patient.

For indications already within the threshold-response class, the cargo problem is largely solved, and the remaining engineering challenge is delivering mRNA efficiently and selectively to the intended cells, particularly beyond the liver [6]. Delivery is necessary but not sufficient: it determines where mRNA can act, not whether it is pharmacologically appropriate to use there (Table 4).

## 14. Triaging Applications by Dose–Response Architecture

The argument of this review can now be operationalized. Instead of inquiring which diseases an mRNA-encoded protein could theoretically address, the field should prioritize candidate indications based on the shape of their dose–response relationship. This shape, rather than the feasibility of expression, determines whether the mRNA’s intrinsic output variability is acceptable. The applications outlined below are categorized according to the five forgiving pharmacological architectures introduced earlier. For each, we highlight how the genuinely suitable uses can be optimized for greater efficiency. Additionally, a sixth category consolidates indications for which the same rationale advises against their consideration.

Threshold-limited enzyme and protein replacement represents the primary and most critical domain. Restoring a fraction of a deficient catalytic or structural protein above a specific threshold suffices to eliminate the phenotype, with any excess remaining inert. The variability of mRNA resides within the benign, flat region of the dose–response curve. Furthermore, mRNA confers advantages not available with recombinant protein or adeno-associated virus, such as the ability to express multiple subunits within a single cell; facilitate routing to intracellular compartments, including the mitochondrion; and permit re-dosing, which is suitable for pediatric patients, where viral vectors are diluted due to liver growth [120,123]. Enhancements in efficiency are achieved via immune-silent, re-dosable lipid nanoparticles that reduce the required dose to exceed the threshold, conformations that extend duration and consequently lengthen dosing intervals, and compartment-targeting sequences that direct the enzyme to its site of action. The class boundary is delineated by any enzyme whose excess proves toxic, thereby reintroducing an upper limit and necessitating output control.

Binary, all-or-none genome modification constitutes the second domain. Given that the therapeutic endpoint is a discrete genomic change, the abundance of the editor determines the kinetics of editing and the proportion of cells edited, rather than the nature of the outcome. Transience is regarded as a safety advantage rather than a liability [147,162]. The transthyretin and CPS1 programs exemplify both efficacy and the rapidity attainable, including a bespoke therapy administered to a single infant within months. Efficiency improvements are achieved through increased in vivo potency, which enhances editing rates at lower exposure levels; ultra-purification processes that maintain the immune-silencing characteristic of editors; and extrahepatic delivery methods that extend editing capabilities beyond the liver. The discipline imposed by the dose–response paradigm aims to identify the minimal editor exposure necessary to attain the target editing rate, as excessive dosing solely escalates off-target risks.

Saturable, receptor-occupancy pharmacology constitutes the third domain. mRNA-encoded antibodies and ligands administered to achieve complete target occupancy demonstrate a plateau effect; consequently, output surpassing the saturating threshold is largely inconsequential, and variability in the upward direction is mitigated [113,114]. This modality eliminates the need for recombinant protein manufacturing and cold-chain logistics while enabling rapid switching between constructs. Efficiency benefits derive from constructs and delivery systems that consistently exceed the saturating level, as well as from formats such as monospecific antibodies with high safety margins that sustain the effective level significantly below any toxic threshold. Caution within this category pertains to potent T cell engagers, whose cytokine saturation point narrows the operational window from above, thereby necessitating fractionation or output regulation.

Integrative immunological priming constitutes the fourth domain, encompassing prophylactic vaccines, personalized and shared-antigen cancer vaccines, and any application whose readout is an adaptive immune response. The immune system integrates antigens produced over a broad range; thus, the absolute output need only fall within a wide window, rendering mRNA variability inconsequential [138,142]. This domain is where the modality already attains success on a large scale. The gains in efficiency are predominantly computational and logistical, including improved neoantigen prediction, expedited per-patient template generation, and formulations that are stable at room temperature, rather than more precise dose control.

Self-limiting and feedback-regulated outputs constitute the fifth domain, encompassing in vivo cell reprogramming, antigen-specific tolerization, and local regenerative factors whose downstream cascades are self-limiting [180,181,182]. Transient, non-integrating expression ensures that reprogrammed cells revert and makes the effect controllable, whereas tolerization relies on the qualitative absence of co-stimulation rather than on the precise quantity of antigen. Non-invasively administered respiratory therapeutics are also relevant in this context when the airway effect remains local and self-limiting, with inhaled, room-temperature-stable mRNA functioning without systemic exposure [187]. Efficiency improvements are achieved through cell-subset targeting, immune silencing, and circuit designs that constrain expression during reprogramming.

The sixth group consists of exposure-controlled applications, which are specific uses that the same analysis recommends avoiding; identifying this group is a fundamental aspect of an honest and transparent program. These applications involve titratable, narrow-therapeutic-index uses where both insufficient and slightly excessive levels of the active agent may cause harm. Clinical practice in such scenarios depends on precise dose adjustments and careful monitoring, rendering them structurally incompatible with a modality that produces the active agent internally within the patient through an unmetered process. Examples include continuous hormone replacement therapy requiring a maintained set point, anticoagulation, narrow-window immunomodulators like those attempted in the IL-2 program, and any indications characterized by a steep bidirectional dose–response relationship. For these applications, the responsible approach is either to refrain from employing the modality or to develop robust closed-loop output control systems prior to their implementation, acknowledging that current control strategies serve to mitigate but do not eliminate the underlying variability.

Two well-known dose-critical proteins establish this boundary as concrete and demonstrate its structural rather than provisional nature. These proteins are illustrative rather than exhaustive; insulin and erythropoietin are highlighted here solely due to their extensive understanding, but they merely exemplify the exposure-controlled class, which also encompasses growth hormone, coagulation factors, numerous cytokines, immunosuppressants maintained within a narrow therapeutic window, and other biologics with limited operational ranges. The discussion pertains to the class, rather than these two specific examples. Insulin presents the clearest case: effective insulin therapy relies on rapid, meal- and glucose-responsive titration within a narrow window, where slight underdosing results in uncorrected hyperglycemia, and slight overdosing causes dangerously low blood glucose levels. mRNA expression involves delays in onset, variable magnitude, sustained output over hours to days, and a lack of straightforward switch-off once delivered, which is contrary to the minute-to-minute controllability that the indication requires. Current mRNA platforms are therefore unsuitable for insulin unless a construct or delivery system demonstrates rapid stop-and-start control, predictable exposure, and clinically acceptable intra- and inter-patient variability, thereby transforming a delayed, variable, and persistent output into a precisely titratable one. In the absence of such a validated control, insulin does not align with current mRNA platforms, and existing approved insulins, defined by specific dose-exposure profiles, cannot be accurately replicated by an mRNA product. Erythropoietin is less sensitive to temporal precision, as it is not titrated on a minute-by-minute basis; however, it remains dose-critical in the relevant sense: sustained overexpression can lead to excessive erythropoiesis and dangerously high hematocrit levels. Therefore, the therapeutic goal is to maintain a consistent level rather than to operate within a supra-threshold range. Variability in erythropoietin output, which is harmless for enzymes functioning on a flat dose–response curve, becomes problematic here because both undershoot and overshoot entail clinical risks. The transient kinetics necessitate repeated dosing to achieve a steady state, which the physiology prefers. Hence, erythropoietin also proves incompatible with current mRNA platforms. This boundary is drawn against currently validated constructs and is deliberately falsifiable rather than permanent. Emerging regulated-expression and synthetic-biology strategies, including small-molecule- or ligand-inducible translational switches, riboswitch- and aptamer-based controllers, engineered negative-feedback and incoherent feed-forward circuits, and glucose- or biomarker-responsive closed-loop designs, are explicitly aimed at converting the open-loop output of mRNA into a controllable one. Should any such system demonstrate rapid start-and-stop control and clinically acceptable intra- and inter-patient variability for insulin or erythropoietin, the corresponding indication would, by the criteria set out here, move from the exposure-controlled to the tractable class. The present assessment, therefore, constrains what today’s constructs can safely do; it does not foreclose what a validated output-control system might achieve. Notably, neither protein is explicitly discussed in the foundational account of the field [6], which itself is indicative, as both are among the most familiar fixed-dose protein therapies in clinical use and are ultimately outside the scope of current modality capabilities. The overarching principle demonstrated by these examples, and emphasized throughout this review, is that the suitability of a platform is determined not by the identity of the protein but by the dose–response architecture of the indication. Where the architecture demands a fixed dose and regimen, delivery and sequence improvements alone are inadequate, absent validated post-delivery output control (Table 5).

Counterarguments and boundaries merit explicit acknowledgment because the classification above pertains to architectures, not to a comprehensive verdict on every molecule. Several qualifications serve to soften the boundaries of the unsuitable category without fundamentally dissolving its essence. An indication that is nominally dose-sensitive may become manageable when a measurable pharmacodynamic biomarker enables low-dose repeat titration, as with a titrated biologic, thereby enabling the correction of between-administration variability against a readout rather than blindly tolerating it. Inducible or feedback-regulated constructs that impose a ceiling on translation can, in principle, transform an uncontrolled output into an effectively saturating one, thereby engineering a more forgiving architecture where the native pharmacology lacks such functionality. Tissue-restricted expression via microRNA-responsive elements effectively eliminates off-target variance, thereby narrowing the effective distribution. Short-lived rescue applications, in which a brief supra-threshold pulse proves therapeutic and the steady-state requirement is not applicable, can also fall within the tractable domain. These approaches are genuine and warrant pursuit, which explains why the boundary is a gradient at its margins rather than an insurmountable barrier. However, none of these modifications alter the fundamental assertion: for a protein whose approved use is defined by a fixed dose and regimen, the residual variability inherent in an in vivo amplification step persists, rendering the product unreproducible. The qualifications expand the conditionally suitable range; they do not convert a genuinely titratable, narrow-index drug into one that resides within it.

## 15. Regulatory and Translational Considerations

The regulatory framework governing mRNA 2.0 diverges from that of vaccines in direct relation to the four frontiers. A reusable systemic therapeutic raises concerns about chronic immunogenicity, anti-drug responses, and cumulative LNP exposure, issues not typically associated with a limited-dose vaccine, thereby emphasizing the importance of demonstrating sustained tolerability over multiple administrations. A genome-editing technology raises distinct concerns about off-target activity and its long-term implications, necessitating the use of sensitive assays to detect rare, unintended single-base or structural alterations, along with extended surveillance to monitor durability and late-onset effects [158,162]. The integration of payload and delivery into a singular product further signifies that two relatively nascent technologies are being concurrently evaluated, thereby amplifying uncertainty, as exemplified by the early safety signals observed with the base editor.

Simultaneously, the modality has demonstrated that regulatory timelines can be significantly reduced when scientific understanding aligns with regulatory agencies’ positions. The initial N-of-1 base-editing therapy progressed from conception to patient administration within approximately six months, with authorization granted for a bespoke product designed for a single infant. This exemplifies a pathway for ultra-rare and personalized therapies that conventional development paradigms are often unable to adequately support [162,163,164]. Similarly, initiatives involving personalized cancer vaccines depend on a regulatory framework that can accommodate a product with a sequence tailored to each patient. These precedents imply that the platform-based nature of mRNA technology, in which the chemistry and manufacturing processes remain largely consistent while the encoded sequence varies, may confer certain regulatory advantages. Specifically, platform-level evidence can expedite the evaluation of individual constructs. Realizing this potential will necessitate continued advancements in standards for characterizing immune silence, purity, and editing fidelity, particularly those tailored for therapeutic applications rather than vaccines.

## 16. Limitations, Risks, and Open Questions

The most fundamental limitation has been identified as the central issue: the dose-to-effect relationship of an mRNA drug is inherently variable because the active agent is synthesized within the patient through translation. This process involves an unregulated amplification step that introduces variability in delivery, escape, and expression. This characteristic is a structural property rather than a transient developmental stage, and, based on current evidence, it precludes the modality from being suitable for narrow-therapeutic-index, titratable indications unless authentic closed-loop output control mechanisms are developed and validated, which remains an unfulfilled objective. All other limitations are subordinate to this primary issue, and the credibility of the field depends upon recognizing this fact rather than suggesting that delivery alone constitutes the sole barrier.

The remaining constraints are tangible yet more manageable. Achieving complete immune silence while maintaining high in vivo potency remains an unresolved optimization challenge, and recent safety signals associated with base editors indicate that combining a nascent payload with an emerging delivery system increases risk [158]. Extrahepatic delivery is primarily in the preclinical stage, and the scalability of ligand- and antibody-conjugated lipid nanoparticle (LNP) production remains uncertain [181]. Cross-species translation presents significant risks, as demonstrated by the Chikungunya-antibody and IL-2-mutein programs, because tolerability in primates does not necessarily ensure human safety at scaled doses; these failures are partly attributed to the dose-control issue, exemplified by a narrow safety margin in humans [115]. Regarding liver targets specifically, many leading indications, such as transthyretin, PCSK9, and lipoprotein(a), can already be effectively addressed with siRNA therapies administered a few times annually. Therefore, genome-editing therapeutics must justify their claim of producing a permanent change over safer, repeatable alternatives [161]. Furthermore, considerations such as off-target editing, duration of effect, late-onset safety concerns, and long-term immune responses require extended surveillance. Although none of these factors are inherently disqualifying, each impacts the timeline, and all are subordinate to the fundamental question of whether the pharmacology of the indication allows for variable outputs without compromising safety.

## 17. Conclusions: A Bounded, and Therefore Credible, mRNA 2.0

The transition from mRNA 1.0 to mRNA 2.0 is indeed significant; however, its scope is characterized by a pharmacological distinction that remains implicitly understood in current discussions. Protein therapeutics should not be regarded as a single category; rather, they are subdivided into exposure-controlled therapeutics, in which clinical benefits depend on delivering a specific amount of active protein according to a prescribed regimen, and threshold-response therapeutics, in which benefits are achieved by surpassing a functional threshold rather than maintaining a precise concentration. Since an mRNA drug provides instructions for intracellular protein synthesis through an amplifying, unregulated translation process rather than directly administering the active protein, the correlation between dose and effect is inherently variable. This variability is not merely a manufacturing or process flaw that can be eliminated by process optimization but a fundamental characteristic of the platform itself. Consequently, mRNA aligns with the threshold-response class: it is naturally compatible with threshold-limited applications such as replacement therapy, binary genome modification, saturable receptor occupancy, integrative immunological priming, and self-regulating or feedback-controlled outputs. Conversely, on current evidence, it remains unsuitable for exposure-controlled therapeutics unless a construct or delivery system demonstrates validated regulation of intracellular output within predefined variability limits.

Within the threshold-response class, prospects are genuinely robust, and the advancements the field is pursuing in delivery, duration control, and purification directly translate into more effective medicines by enhancing potency and reliability where the underlying pharmacology already tolerates residual variability. For the exposure-controlled class, delivery or sequence optimization alone does not render mRNA a viable option, and where such a protein is already approved with a fixed dose and regimen, current mRNA platforms cannot replicate its exposure profile, absent demonstrated post-delivery output control. The operational message, therefore, is a classification rule rather than a promise regarding delivery: candidate indications should be triaged by class prior to development, prioritizing threshold-response cases and excluding exposure-controlled cases unless post-delivery output control has been demonstrated. Delivery determines the feasible applications of mRNA; pharmacology determines its appropriateness. When applied with this discipline, the distinction enhances the platform’s credibility by directing the technology towards indications for which it is uniquely suited and declining those for which it is not. The crucial point is that the classification is intended to be applied prospectively, at the point of target selection, rather than retrospectively after a program has failed in clinical trials. Used proactively, it functions as a practical filter that conserves resources and protects patients by excluding exposure-controlled indications prior to development; used only in hindsight, it merely elucidates failures that the framework could have predicted. Therefore, the next era of mRNA medicines should be strategically planned from the outset, taking into account the target’s dose–response architecture.

## Figures and Tables

**Table 3:** Comparison of linear, circular, and self-amplifying RNA conformations.

Attribute	Linear Modified mRNA	Circular RNA	Self-Amplifying RNA
Topology	Cap, 5′ UTR, coding sequence, 3′ UTR and poly(A) tail, with free ends	Covalently closed loop with no free ends; translation initiated internally rather than cap-dependently	Linear transcript encoding an alphavirus replicase complex together with the gene of interest
Expression kinetics	Rapid onset with a short-lived pulse, typically on the order of 24 to 72 h	Broader and more sustained output than linear mRNA of the same sequence	Delayed onset, high amplitude and prolonged duration following intracellular amplification
Dose requirement	Standard	Potentially lower through extended output per transcript	Substantially lower, since the template is amplified in the cell
Innate immunogenicity	Lowest of the three when modified nucleosides and ultra-purification are used	Does not depend on modified nucleosides; requires control of nicked species and ligation byproducts	Higher, because double-stranded replication intermediates are generated during amplification
Principal limitation	Short duration limits chronic replacement without frequent re-dosing	Manufacturing complexity of circularization and purification	Large construct size complicates packaging and delivery; innate activation is harder to suppress
Best-suited use	Genome editing and other applications where a brief pulse is an advantage; most current clinical programs	Applications requiring sustained expression from a single administration	Prophylaxis and other settings that tolerate or benefit from innate activation
Clinical status	All approved mRNA products and the majority of clinical-stage programs	First-in-human Phase 1/2a (RXRG001, radiation-induced xerostomia) [91]	Approved as zapomeran (Kostaive) [20]

The conformation is selected according to the duration required by the indication rather than applied uniformly. UTR, untranslated region.

**Table 4:** Representative clinical-stage mRNA candidates, development status, and assignment under the Dose–Response Architecture Classification.

Candidate	Indication	Sponsor	Status as of July 2026	Architecture and Assignment
AZD8601	Regenerative angiogenesis during coronary artery bypass grafting	AstraZeneca and Moderna	Phase 2 completed; safety and tolerability endpoints met [101]	Threshold-limited and self-limiting; suitable
mRNA-6231	Autoimmune disease (Treg-selective IL-2 mutein)	Moderna	Discontinued on undisclosed trial data [6]	Steep and bidirectional; exposure-controlled and unsuitable
mRNA-3927	Propionic acidemia	Moderna	Phase 1/2; 70 percent reduction in decompensation risk in a subset [120]	Threshold-limited replacement; suitable
mRNA-3705	Methylmalonic acidemia	Moderna	Phase 1/2 (NCT04899310) terminated; open-label extension (NCT05295433) recruiting [124,125]	Threshold-limited replacement; suitable
mRNA-3745	Glycogen storage disease type 1a	Moderna	Not advancing to Phase 2 [129,130,131]	Threshold-limited replacement; suitable
ARCT-810	Ornithine transcarbamylase deficiency	Arcturus	Phase 2; positive interim multiple-dose data [135]	Threshold-limited replacement; suitable
ARCT-032 (inhaled)	Cystic fibrosis	Arcturus	Phase 2 interim data reported [187,188]	Threshold-limited and local; suitable
RCT1100 (inhaled)	Primary ciliary dyskinesia	ReCode	Phase 1b; initial evidence of biological activity [189]	Threshold-limited and local; suitable
Nexiguran ziclumeran (NTLA-2001)	Transthyretin amyloidosis	Intellia	Phase 3 MAGNITUDE and MAGNITUDE-2; clinical holds lifted in 2026 and enrollment resumed [148,149,150,151]	Binary genome modification; suitable
VERVE-102	PCSK9 base editing for LDL cholesterol lowering	Verve	Phase 1b clinical proof of concept [156]	Binary genome modification; suitable
BEAM-301	Glycogen storage disease type 1a	Beam	Early development; up to 60 percent correction in rodent models [132]	Binary genome modification; suitable
CPS1 N-of-1 base editor	Carbamoyl-phosphate synthetase 1 deficiency	Academic and industry consortium	Single-patient authorization; reduced ammonia and improved protein tolerance [162,164]	Binary genome modification; suitable
Intismeran autogene (mRNA-4157) with pembrolizumab	Resected melanoma and other solid tumors	Moderna and Merck	Phase 3 ongoing; 49 percent reduction in recurrence or death at median five-year Phase 2b follow-up [138,139]	Integrative immune priming; suitable
Autogene cevumeran	Pancreatic ductal adenocarcinoma	BioNTech and Genentech	Phase 2; functional T cells persisting up to four years [141,142]	Integrative immune priming; suitable
BNT142	Claudin-6-positive solid tumors (CLDN6 and CD3 bispecific)	BioNTech	Phase 1/2; early activity with dose-dependent cytokine elevation [113,114]	Saturable with a cytokine ceiling; conditionally suitable
mRNA-1944	Chikungunya virus (CHKV-24 IgG)	Moderna	Phase 1; not advanced owing to tolerability at scaled doses [115]	Saturable receptor occupancy; conditionally suitable
Descartes-08	Generalized myasthenia gravis (autologous mRNA CAR-T)	Cartesian	Phase 3 AURORA enrolling; lupus development paused [176,177,178,179]	Self-limiting cell reprogramming; suitable
RXRG001	Radiation-induced xerostomia (circular RNA)	RiboX	First-in-human Phase 1/2a [91]	Threshold-limited and local; suitable

Status is drawn from the sources cited in the preceding sections and from company and registry disclosures accessed 23 July 2026. Assignment follows the criteria in Section 4.2 and the decision sequence in Figure 3. The pattern is that programs in the threshold-response class have advanced, whereas the one clearly exposure-controlled program in this table, the IL-2 mutein, did not. CAR, chimeric antigen receptor; LDL, low-density lipoprotein.

**Table 5:** Triage of representative applications by dose–response architecture.

Example Application	Dose–Response Architecture	Classification	Basis
Enzyme replacement (for example, propionic and methylmalonic acidemia)	Threshold-limited; flat above a catalytic threshold	Suitable	Supra-threshold output is inert; variability lands on the plateau
Genome and base editing	Binary, all-or-none; permanent discrete endpoint	Suitable	Editor abundance sets kinetics, not the final state; transience is an asset
Cancer vaccines (personalized and shared-antigen)	Integrative immune priming over a wide window	Suitable	Readout is an integrated immune response, not a protein level
In vivo CAR reprogramming and antigen-specific tolerization	Self-limiting; downstream cellular and immune feedback	Suitable	Effect is regulated downstream; transient non-integrating expression reverts
Therapeutic antibodies (monospecific)	Saturable receptor occupancy with a high safety ceiling	Conditionally suitable	Plateau absorbs upward variability if output reliably exceeds saturation
Bispecific T cell engagers	Saturable but with a cytokine ceiling close to the effective level	Conditionally suitable	Narrow upper bound; needs fractionation or output control
IL-2 and narrow-window immunomodulators	Steep, bidirectional; effect inverts above a narrow band	Unsuitable	Treg selectivity is lost above the window; the mRNA-6231 program was discontinued
Erythropoietin	Held-level target; overshoot raises hematocrit dangerously	Unsuitable	Both undershoot and overshoot carry cost; aim is a held level
Insulin	Narrow-index, real-time titratable to glucose	Unsuitable	Delayed, variable, persistent output is the inverse of titratability

Classification reflects the dose–response architecture of the indication rather than the feasibility of expressing the protein. Suitable and conditionally suitable entries are threshold-response therapeutics: suitable, the indication tolerates supra-threshold variability; conditionally suitable, suitable only if output reliably exceeds a saturating level or is fractionated or controlled. Unsuitable entries are exposure-controlled therapeutics, whose safe use depends on a defined, titratable exposure that an unmetered translation step cannot provide.

## Data Availability

No new data were created or analyzed in this study. Data sharing is not applicable to this article. The published literature, clinical trial registry records, and company disclosures discussed in the review are cited in the reference list.
